# A Novel *Dnmt3a1* Transcript Inhibits Adipogenesis

**DOI:** 10.3389/fphys.2018.01270

**Published:** 2018-10-02

**Authors:** Bahareldin A. Abdalla, Zhenhui Li, Hongjia Ouyang, Endashaw Jebessa, Tianhao Sun, Jia-ao Yu, Bolin Cai, Biao Chen, Qinghua Nie, Xiquan Zhang

**Affiliations:** ^1^Department of Animal Genetics, Breeding and Reproduction, College of Animal Science, South China Agricultural University, Guangzhou, China; ^2^National-Local Joint Engineering Research Center for Livestock Breeding, Guangzhou, China; ^3^Guangdong Provincial Key Lab of Agro-Animal Genomics and Molecular Breeding, the Key Lab of Chicken Genetics, Breeding and Reproduction, Ministry of Agriculture, Guangzhou, China

**Keywords:** *Dnmt3a1* transcript, *Dnmt3a*, expression, aging, high-fat diet, preadipocytes proliferation, early differentiation

## Abstract

*DNA (cytosine-5)-methyltransferase 3a* (*Dnmt3a*) is an enzyme that catalyzes the transfer of methyl groups to specific CpG forms in DNA. In mammals, two variant transcripts of *Dnmt3a* have been successfully identified. To the best of our knowledge, no *Dnmt3a* transcripts in an avian have been successfully identified. This study was performed to detect different transcripts of *Dnmt3a* in chickens and to examine whether a novel *Dnmt3a* transcript named *Dnmt3a1* may regulate adipogenesis. In addition to cloning, sequencing, transcript detection, and expression studies, a novel *Dnmt3a1* transcript overexpression and knockdown were conducted to explore the potential role of *Dnmt3a1* in preadipocyte proliferation and the early stage of adipocyte differentiation. In chicken abdominal fat tissue, we detected a novel *Dnmt3a1* transcript that differs from *Dnmt3a* by lacking 23 amino acids at the exon-1/exon-2 border. *Dnmt3a1* mRNA was ubiquitously expressed in a variety of tissues or cells and highly expressed in chicken adipose tissue/cells. The expression of *Dnmt3a1* was regulated under different physiological conditions including aging, fasting, and high-fat diet. In addition, overexpression of *Dnmt3a1* significantly decreased preadipocyte proliferation and induced cell-cycle arrest while its inhibition increased cell proliferation and S-phase cells. Furthermore, the overexpression of *Dnmt3a1* significantly upregulated the mRNA level of cell-cycle-related genes, such as *CDKN1A*, *CDKN1B*, *CCNB3*, *CCND2*, *CCNG2*, *CDKN2B*, and *CDK9*, or the protein level of CDKN1A, CDKN1B, and CCNG2. Conversely, the knockdown of *Dnmt3a1* by siRNA had the opposite effects. Moreover, during early adipocyte differentiation, the overexpression of *Dnmt3a1* significantly decreased the mRNA and the protein levels of PPAR-γ, C/EBP-α, ADIPOR1, and STAT3, and the mRNA levels of *FAS*, *LEPR*, *LPL*, *PRKAB2*, and *ATGL.* In contrast, their expression was significantly increased after the knockdown of *Dnmt3a1*. Taken together, we identified a novel transcript of *Dnmt3a*, and it played a potential role in adipogenesis.

## Introduction

Adipogenesis is a process of cell differentiation by which preadipocytes turn into or display the adipocyte phenotype. Adipose tissue is an important site for lipid storage and comprises a variety of cell types, such as connective tissue matrix, nerve tissue, immune cell, stromovascular cell, stem cell, mesenchymal precursor, preadipocytes, and adipocytes, which together contribute to its role as an endocrine organ (Gregoire et al., 1998; Kershaw and Flier, 2004). Adipose tissue/cell development processes can be controlled by many genetic regulators (Barsh et al., 2000; Abdalla et al., 2018) that are governed by complex molecular regulatory networks. Many cell-cycle-related genes or proteins have been shown to be involved in adipogenesis *in vitro* and/or *in vivo*, such as *cyclin-dependent kinase inhibitor 1A* (*CDKN1A*; also known as *p21*), *cyclin-dependent kinase inhibitor 1B* (*CDKN1B*; also known as *p27*), *cyclin G2* (*CCNG2*), and *cyclin-dependent kinase 9* (*CDK9*) (Lin et al., 2003; Nam et al., 2008; Aguilar et al., 2010). In addition, *peroxisome proliferator-activated receptor gamma* (*PPAR-γ*) and *CCAAT/enhancer-binding protein alpha* (*C/EBP-α*) are reported to play the most prominent functions in fundamental cellular processes including the signaling system that assesses the terminal differentiation of adipose tissue/cell development (Brun et al., 1996; Lefterova et al., 2014). Obesity is a common disorder encountered in clinical practice and related to many diseases, such as cancer, hypertension, atherosclerosis, and type-2-diabetes (Flegal et al., 2007; Alvehus et al., 2010). Obesity occurs when the body consumes more calories than it expends and in turn reserves too much adipose tissue or fat (Bray and Popkin, 1998). Controlling or inhibiting preadipocyte proliferation and/or adipocyte differentiation might eventually lead to a way to treat or prevent obesity and/or obesity-related diseases (Min et al., 2013). The most extensive research in preadipocyte cell lines such as mouse 3T3-L1 and 3T3-F442A has focused on the effects of transcription factors/genes on cell lineage determination and terminal differentiation. However, the biological function of many variant transcripts of genes during primary preadipocyte proliferation and early differentiation remain to be investigated.

*DNA (cytosine-5)-methyltransferase 3a* (*Dnmt3a*) is an enzyme that catalyzes the transfer of methyl groups to specific CpG forms in DNA and the process is known as DNA methylation. The enzyme is encoded by the *Dnmt3a* gene in chicken. *Dnmt3a* belongs to the family of enzymes named *DNA methyltransferases* (*Dnmts*), which consists of *Dnmt1*, *Dnmt3a*, and *Dnmt3b* (Okano et al., 1998; Okano et al., 1999). In mice, both *Dnmt3a* and *Dnmt3b* have important roles in embryonic stem cell development and are required for genome-wide *de novo* methylation (Okano et al., 1999). It has been shown that eukaryotic *Dnmt1*, *Dnmt3a*, and *Dnmt3b* are expressed in most types of cell division (reviewed in Goll and Bestor, 2005). *Dnmt3a* expression accelerates from a low level on 6.5 d during germ cell development to a moderate level at 25 weeks of age in the gonads of male and female chickens (Rengaraj et al., 2011). Previous work showed that the expression of *Dnmt3a* was markedly upregulated in the adipose tissue of obese mice (Kamei et al., 2010). In wild-type mice and transgenic mice overexpressing *Dnmt3a* treated with a high-fat diet, the body weight gain did not alter significantly, but the expression of *tumor necrosis factor*-α (*TNF-alpha*) and *monocyte chemoattractant protein-1* (*MCP-1*) were higher in transgenic mice overexpressing *Dnmt3a* than in wild-type mice (Kamei et al., 2010).

In mammals, some variant transcripts of *Dnmts* have been identified in healthy or cancerous tissues/cells (Mertineit et al., 1998; Okano et al., 1998; Robertson et al., 1999; Xie et al., 1999; Chen et al., 2002; Saito et al., 2002; Weisenberger et al., 2002). However, the functional consequence of the variant transcripts of *Dnmts* is still poorly understood. Using Northern blot analyses, two variant transcripts of *Dnmt3a* have been detected in mouse and human tissues/cells (Okano et al., 1998; Robertson et al., 1999; Xie et al., 1999). The murine RNA has two variant transcripts of *Dnmt3a* (reviewed in Yang et al., 2015). The *Dnmt3a2* isoform has been identified in humans and mice by Chen et al. (2002). They found that *Dnmt3a2* is a shorter isoform of *Dnmt3a* and is the predominant type in embryonic stem cells and embryonal carcinoma cells, and it can also be found in testis, ovary, thymus, and spleen. To our knowledge, no published study is available regarding chicken *Dnmt3a* transcripts. As we have identified the *Dnmt3a1* transcript from the abdominal fat of chicken, we hypothesize that it is also involved in adipogenesis in avians; therefore, the present study was performed to identify the potential role of *Dnmt3a1* in adipogenesis by using chicken as a model species to expand our knowledge to the non-mammalian species.

## Materials and Methods

### Ethics Statement

All the experimental procedures in this study were conducted under a protocol approved by the Animal Ethics Committee of the Institutional Animal Care and Use Committee in the College of Animal Science, South China Agricultural University (SCAU), Guangdong province, China, with approval number SCAU#0011.

### Animals and Tissue Collection

A total of 36 (12 females at 16 weeks of age; 6 females at 21 weeks of age; 18 females at 24 weeks age) Xinghua (XH) chickens (a Chinese local breed with a slow growth rate) were reared in individual cages at the chicken farm of SCAU with a 12-h light/dark cycle. To determine the *Dnmt3a1* tissue expression profile, the six 21-week-old females were slaughtered and 14 different tissue/organ samples were rapidly collected, including abdominal fat, pituitary, subcutaneous fat, cerebellum, brain, hypothalamus, spleen, ovary, lung, kidney, liver, heart, leg muscle, and breast muscle. To study the effect of the feeding status on the *Dnmt3a1* expression profile in the hypothalamus, liver, subcutaneous fat, and abdominal fat, the 24-week-old females were fed *ad libitum*, fasted for 3 days, or fasted for 3 days followed by refeeding for 1 day (*n* = 6/case). To obtain high-body-weight samples, the remaining 16-week-old females were divided into two groups (*n* = 6 in each group) and fed a high-fat diet consisting of 40% carbohydrate, 25% fat, and 20% protein or fed a standard chicken diet (control group) (41% carbohydrate, 5% fat, and 22% protein) for 3 weeks, and the samples were collected from all chickens.

Another 24 females from the fifth generation of the Guangxi three-yellow commercial broiler aged 3, 14, 26, and 40 weeks (*n* = 6/age) were obtained from Guangzhou Kwangfeng Industrial Co., Ltd. (Guangzhou, China) and used to examine the effect of aging on the *Dnmt3a1* expression profile in the abdominal fat, subcutaneous fat, liver, and leg muscle of the chickens. All birds had free access to fresh and clean water *ad libitum*. The tissue samples collected were immediately frozen in liquid nitrogen and stored at −80°C until use.

### 5′- and 3′-Rapid Amplification of Complementary DNA Ends (RACE)

A BD SMART^TM^ RACE cDNA Amplification Kit (BD Biosciences Clontech, United States) was used for performing 5′-RACE PCR, while a SMARTer^®^ RACE 5′/3′ Kit (Clontech, Japan) was used for performing 3′-RACE PCR, following the manufacturer’s protocols. The partial chicken *Dnmt3a* complementary DNA (cDNA) sequence was obtained from GenBank accession No: NM_001024832.1. The primer sets used for 5′-RACE and 3′-RACE are presented in **Table 1**. RACE PCR amplification was carried out to obtain the full-length cDNA sequence of the chicken *Dnmt3a*. The total RNA from chicken abdominal fat tissue was used as a template for nested-PCR reactions in 5′- and 3′-RACE, and a step-down PCR was used; the reaction profile was 2 min at 94°C followed by 5 cycles of 98°C for 10 s and 74°C for 3 min; then 5 cycles of 98°C for 10 s and 72°C for 3 min; then 5 cycles of 98°C for 10 s and 70°C for 3 min; then 25 cycles of 98°C for 10 s and 68°C for 3 min; followed by a 8-min final extension at 68°C. The products of the 5′- and 3′-RACE PCR were detected by gel electrophoresis in 1.5% (w/v) agarose gel stained with ethidium bromide, and then PCR bands were recovered from the gel (purified) and cloned into the pJET 1.2/blunt cloning vector using a CloneJET PCR Cloning Kit (Fermentas, United States) following manufacturer’s recommendations and sequenced by Sangon Biotech (Shanghai, China) or TsingKe Biological Technology (Beijing, China).

**Table 1:** Primers used for RACE PCR, *Dnmt3a* variant transcript, and genomic DNA detection of *Dnmt3a1* deleted sequence.

Primer name	Forward (F) and reverse (R)	Sequence 5′ to 3′ direction	Annealing temperature (°C)
Dnmt3a-5′-RACE-Outer primer		AAGCAGTGGTATCAACGCAGAGT	Step-down PCR
Dnmt3a-5′-RACE-Inner primer		TCCTTGCTTTTCTCTGTGGCCT	Step-down PCR
Dnmt3a-3′-RACE-Outer primer		CAAAGACCAGCACTTCCCCGTC	Step-down PCR
Dnmt3a-3′-RACE-Inner primer		GGCAGCCCCTGCAACGACCTCTCTATAGTG	Step-down PCR
Dnmt3a-variant transcript	F	TGCGCCATGGTGGAAAGCAGTGACACACCC	66
detection	R	GGCACAGTCCCCGTCCGGCTCTCCTA	
Dnmt3a1-deleted sequence	F	AGGAGGACGAGACGGAGAGCC	67
genomic DNA detection	R	ACAGCTACGCACACACACACTTGCC	

### Transcript Variant Detection and Construction of *Dnmt3a1* Overexpression Plasmid

#### Transcript Variant Detection of *Dnmt3a* Gene

Ten 3-week-old chicks of Guangxi three-yellow chickens were obtained from Guangzhou Kwangfeng Industrial Co., Ltd. (Guangzhou, China) and used for extracting total RNA from abdominal fat tissue (shown below). The extracted total RNA was used as a template for PCR analysis of *Dnmt3a* transcript variants, and was reverse transcribed into cDNA using the PrimeScript RT Reagent Kit with a gDNA Eraser (Perfect Real Time) (TaKaRa, Japan), following the manufacturer’s instructions. The primer pair used to amplify the full-length coding sequence of chicken *Dnmt3a* for detecting *Dnmt3a* transcript variants is listed in **Table 2** and synthesized by Sangon Biotech. PCR amplification of each cDNA was performed in a volume of 50 μL using PCR KOD FX reagents (Toyobo Life Science Department, Japan) following manufacturer’s instructions. The thermal cycler conditions were: predenaturation for 2 min at 94°C, followed by 36 cycles of denaturation at 98°C for 10 s, annealing at 66°C for 30 s and extension at 68°C for 3 min, and finally extension at 68°C for 7 min. All PCR products were evaluated by 1.5% agarose gel electrophoresis. All bands detected in PCR were directly cloned into the pSDS-25202 vector (SiDanSai ^1^; Shanghai, China) and/or the pJET 1.2/blunt cloning vector, and sequenced by Sangon Biotech or TsingKe Biological Technology. VecScreen online software (NCBI) was used to screen the sequences for vector contamination. The National Center for Biotechnology Information’s Basic Local Alignment Search Tool (BLAST) analysis was performed to confirm the specificity of the sequence and map its position.

**Table 2:** Sequences of primer pair used to construct *Dnmt3a1* overexpression plasmid (pSDS-Dnmt3a1) and siRNA (si-Dnmt3a1) targeting *Dnmt3a1.*

Name	Target sequence
pSDS-Dnmt3a1	Sense: 5′-GGGGggtctctagtgTGCGCCA TGGTGGAAAGCAGTGACACACCC-3′
	Anti-sense: 5′-GCCGggtctcgtgggGGC ACAGTCCCCGTCCGGCTCTCCTA-3′
si-Dnmt3a1	Sense: 5′-GCCGUCGACAAGAACACAU dTdT-3′
	Anti-sense: 3′-dTdT CGGCAGCUGUUCUUGUGUA-5′

*Sequences in small letters represent the enzyme cutting sites.*

#### Construction of *Dnmt3a1* Overexpression Plasmid

The plasmid expressing *Dnmt3a1* was constructed to contain a full-length chicken *Dnmt3a1* coding sequence. Briefly, the primer pair pSDS-Dnmt3a1 (**Table 2**) synthesized by Sangon Biotech was used to amplify the *Dnmt3a* coding sequence and generate the overexpression plasmid. The PCR product from the chicken abdominal fat tissue total RNA containing the *Dnmt3a1* transcript (after sequence confirmation) was cloned into the expression vector pSDS-25202 (SiDanSai) by digesting the PCR product with the BsaI restriction enzyme and ligating the fragment to produce pSDS-Dnmt3a1, and then sequenced by TsingKe Biological Technology for validation. The pSDS-25202 empty vector was used as negative control and named pSDS-NC. For confirming the *Dnmt3a1* overexpression efficiency, preadipocytes were seeded into 6-well or 12-well culture plates and transfected with pSDS-Dnmt3a1 and pSDS-NC. At 48 h after the initial transfection, cells were harvested and immediately subjected to total RNA or protein isolation, and quantitative real-time PCR (qRT-PCR) and Western blotting were performed for confirming the overexpression efficiency.

### Small Interfering RNA (siRNA)

The siRNA target sequence used for *Dnmt3a1* knockdown (si-Dnmt3a1) is presented in **Table 2**. The si-Dnmt3a1 consisted of two complementary 19 nucleotide RNA strands (the sense and antisense strands) with 3′-deoxythymidine dinucleotide (dTdT) overhangs. A sequence-scrambled siRNA [as negative control (si-NC; sequence not shown)] was used to ensure specificity. All siRNAs were purchased from RiboBio (Guangzhou, China). Chicken primary preadipocytes were seeded into 12-well culture plates and transfected with si-Dnmt3a1 and si-NC. At 48 h post-transfection, cells were harvested and immediately subjected to total RNA and protein isolation. For confirming the *Dnmt3a1* knockdown efficiency, qRT-PCR and Western blot analyses were performed on the isolated total RNA and protein, respectively.

### Primary Preadipocyte Preparation and Culture

Chicken primary preadipocytes were isolated and then cultured to determine the function of *Dnmt3a1* in chickens. Primary preadipocytes were prepared as described previously (Ramsay and Rosebrough, 2003) with the following modifications. In brief, abdominal fat tissue was collected from 3-week-old chicks of the Guangxi three-yellow chicken (Guangzhou Kwangfeng Industrial Co., Ltd.) under sterile conditions. DMEM/F12 (Gibco, United States) supplemented with 15% (v/v) fetal bovine serum (Gibco) and 0.8% antibiotic–antimycotic (anti–anti) 100× containing streptomycin sulfate and penicillin G sodium (Life Technologies, United States) was used as the growth culture medium. Cells were cultured at 37°C in a 5% CO_2_, humidified atmosphere. The primary preadipocytes were seeded and proliferated. After the isolated cells were cultured for two passages (5–6 days) and once cells reached 90–100% confluence (day 0), the growth medium was removed and the cells were fed a lower serum medium containing DMEM/F12, 2% horse serum (Gibco, New Zealand) and 0.5% anti–anti 100 × , and the medium was changed every 2 days to induce differentiation. On day 8, the induction of complete adipocyte differentiation was confirmed by measuring the exogenous and endogenous lipid droplet accumulation in oil red O staining.

### RNA Extraction, cDNA Synthesis, and qRT-PCR Analysis

Total RNA was extracted from each tissue or cell using RNAiso reagent (TaKaRa) according to manufacturer’s instructions. The total RNA isolated from the tissue or cell was reverse-transcribed into cDNA by using a PrimeScript RT Reagent Kit with a gDNA Eraser (Perfect Real Time) (TaKaRa) as recommended by the manufacturer. For quantitative measurement, mRNA was quantified with iTaq^TM^ Universal SYBR Green Supermix (Bio-Rad Laboratories, United States) following the manufacturer’s protocol in a QuantStudio^TM^ 5 Real-Time PCR Instrument (96-Well 0.2 ml Block) (Thermo Fisher Scientific, United States). Primers used for qRT-PCR (see **Table 3**) were designed by Premier Primer 5.0 Software (PREMIER Biosoft International, Palo Alto, CA, United States) or OLIGO Primer Analysis Software v. 7 (Molecular Biology Insights, Inc., Vondelpark Colorado Springs, CO, United States), and synthesized by Sangon Biotech or TsingKe Biological Technology. The gene expression was normalized in all samples to the chicken glyceraldehyde 3-phosphate dehydrogenase (*GAPDH*) gene. The optimum thermal cycler conditions were: 95°C for 3 min, followed by 40 cycles at 95°C for 15 s, X°C (see **Table 3** for annealing temperature) for 40 s, 95°C for 15 s and 60°C for 1 min. The relative levels of the gene expression data were calculated using the comparative 2^−ΔΔCT^ method (Livak and Schmittgen, 2001).

**Table 3:** Primers used for qRT-PCR.

Target gene symbol	F&R	Sequence 5′ to 3′ direction	Accession no.	Annealing temperature (°C)	Amplicon length (bp)
*Dnmt3a1*	F	GATGAGGGCAAGGAGGAGAAG	LC379990	58	314
	R	TCGGTGGGCTGCTGTGAG			
*Dnmt3a*	F	TGAGCTGGCCCCTTCCGACCC	LC381635	58	349
	R	GCTGGCCTCCTCCTCCTTCTG			
*CDKN1A*	F	GAGAAGAGTTGTCCACGATAAGCC	NM_204396.1	58	252
	R	CCATTCCAGTCCTCCTCAGTCC			
*CDKN1B*	F	TCGCTGTGCTGGGCTGAA	NM_204256.2	58	212
	R	CAAGGACGAAAGGATGTGGG			
*CCNB3*	F	CCCCACGCTGGAGTATTA	NM_205239.2	58	149
	R	TGGCGACCTCAAAGAAGA			
*CCND2*	F	CCGAACTTGCTCTACGACG	NM_204213.1	58	217
	R	GCTCAATGAAATCATGTGGG			
*CCNG2*	F	TGCCAACAATACCAGAGG	XM_420475.5	58	260
	R	TACAGAATACCACAATCCC			
*CDK9*	F	AAACCCACCCGCTCTTCCC	NM_001006201.2	58	252
	R	CTGGTGCTGCTCCGTGTT			
*GADD45b*	F	CGCTTCGGTCCCTTTGGTGA	XM_015299957.1	58	198
	R	CCGGCAGTTGTTGTGCAGTCAG			
*PPAR-γ*	F	CTCCTTCTCCTCCCTATTT	NM_001001460.1	60	227
	R	TTTCTTATGGATGCGACA			
*C/EBP-α*	F	GACAAGAACAGCAACGAGTACCGC	NM_001031459.1	56	195
	R	CCTGAAGATGCCCCGCAGAGT			
*ADIPOR1*	F	GGAGAAGGGCGAGCAGGGAG	NM_001031027.1	60	110
	R	GCAGAGGCAGCGTCAGCACA			
*FABP4*	F	GCCTAATTTAACTATCAGCA	NM_204290.1	60	222
	R	CCATCCACCACTTTTCTC			
*FAS*	F	CGCAGGCATAGCAGGAAA	NM_205155.2	60	180
	R	CCAAAGAAGGAGGCATCAA			
*FTO*	F	AGGGCAAACTTCCATACC	NM_001185147.1	60	215
	R	ACGAGACACTGGGGTAAA			
*LEPR*	F	CCAACCCTTCCTTGCTAA	NM_204323.1	60	182
	R	GCCTTCAACCCAACATTC			
*LPL*	F	CCAAGGTAGACCAGCCATTC	NM_205282.1	60	154
	R	TGCTCCAGGCACTTCACA			
*PRKAB2*	F	AAGGAGTTTGTTTCGTGGCA	NM_001044662.1	58	189
	R	TGGGAGGTCCAGGATAGCG			
*STAT3*	F	CCATCCTGAGCACCAAGC	NM_001030931.1	58	174
	R	TGATTTCCGCAAACGACA			
*ATGL*	F	TGTCCAAAGAAGCACGAAA	NM_001113291.1	58	255
	R	GAGGTATCAGCCCACAGTAGA			
*VLDLR*	F	GAGTCCCAGTTCCAGTGTAGT	NM_205229.1	58	208
	R	TCTCGTCAGACCCATCCT			
*GAPDH*	F	CAACTTTGGCATTGTGGAGG	NM_204305.1	52–67	130
	R	CGCTGGGATGATGTTCTGG			

### Genomic DNA Extraction and Global 5-Methylcytosine (5-mC) Analysis

The genomic DNAs from chicken primary preadipocytes were isolated 48 h after transfection with pSDS-Dnmt3a1 and respective negative control by E.Z.N.A^®^ Tissue DNA Kit-cultured cell protocol (Omega Bio-Tek, United States) following the supplier’s recommendations. Approximately 65 ng of genomic DNA was used for DNA global methylation analysis performed with the 5-mC DNA ELISA Kit (Zymo Research, United States) according to the manufacturer’s instructions. The absorbance was measured at 405 nm using a Synergy^TM^ Neo2 Multi-Mode Reader (BioTek, United States). Raw values were quantified and methylation levels (% 5-mC) were estimated using a standard curve of the methylated DNA standard provided by the manufacturer. Values are presented as methylation percent relative.

### Western Blot Analysis

Western blot analysis was carried out using a standard protocol. Chicken primary preadipocytes were seeded in 6-well culture plates, once the cells reached 80% confluence or on day 2 during the differentiation induction of preadipocytes, and transfection was performed with pSDS-Dnmt3a1 or pSDS-NC and si-Dnmt3a1 or si-NC (as control) using Lipofectamine 3000 (Invitrogen, Carlsbad, CA, United States) according to the manufacturer’s protocol. At 48 h post-transfection, the cells were harvested, washed twice with phosphate-buffered saline (PBS), and lysed using 1× radioimmunoprecipitation assay buffer containing 1× protease inhibitor cocktail (Beyotime Biotech, Shanghai, China). Cell lysates were cleared by centrifugation at 13,000 rpm for 9 min at 4°C. Protein concentrations were determined using the Pierce^TM^ BCA Protein Assay Kit (Thermo Fisher Scientific). The following primary antibodies were used: anti-Dnmt3a (ab16704; Abcam, United Kingdom), anti-CDKN1A/p21 (GTX112898; GeneTex, United States), anti-CDKN1B/p27 (bs-0742R; Bioss, China), anti-CCNG2 (GTX77670; GeneTex), anti-PPAR-γ (NB120-19481; Novus Biologicals, Littleton, CO, United States), anti-C/EBPα (70R-49534; Fitzgerald, United States), anti-ADIPOR1 (ab77611; Abcam), and anti-STAT3 (610189; BD Biosciences, United States). GAPDH (1A6) monoclonal antibody-HRP (MB001H; Bioworld Technology, Inc., United States) was used to ensure equal sample loading control. The goat antimouse IgG(H & L)-HRP (BS12478; Bioworld Technology, Inc.) and goat antirabbit IgG(H & L)-HRP (BS13278; Bioworld Technology, Inc.) were used as a secondary antibody. The intensity of bands was quantified with ImageJ software.

### Cell Counting Kit-8 (CCK-8) Assays

Preadipocytes were seeded in a 96-well culture plates and at 50% confluence. They were transfected with pSDS-Dnmt3a1 or pSDS-NC or with si-Dnmt3a1 or si-NC using Lipofectamine 3000 (Invitrogen). Cell proliferation was monitored daily for 6 days (day 0, day 1, day 2, day 3, day 4, and day 5) using TransDetect CCK-8 kit (TransGen Biotech, Beijing, China). Exactly each 24 h, 10 μL per well of CCK-8 solution was added and the optical density (OD) was measured at 450 nm after a 1 h incubation (at 37°C) using a Synergy^TM^ Neo2 Multi-Mode Reader (Bio-Tek).

### EdU Incorporation Assay

EdU assays were performed in proliferating preadipocytes following a standard protocol. Briefly, 24 h after transfection with pSDS-Dnmt3a1, pSDS-NC, si-Dnmt3a1 or si-NC, the preadipocytes were incubated with 10 μM 5-ethynyl-2′-deoxyuridine (EdU; RiboBio) for 24 h at 37°C, then 1× Apollo reaction cocktail (RiboBio) was added to the cells and incubated for 30 min. The proliferating cells were visualized under a Leica DMi8 fluorescent microscope using the images of randomly selected fields obtained from the fluorescence microscope. The preadipocyte proliferation rate was determined by the numbers of EdU-stained cells (red) normalized to the numbers of Hoechst 33342-stained cells (blue).

### Cell-Cycle Assays

Cells were seeded in 12-well culture plates and transfected with pSDS-Dnmt3a1, pSDS-NC, si-Dnmt3a1 or si-NC using Lipofectamine 3000 (Invitrogen). After 48 h of transfection, cells were harvested and fixed in 70% ethanol overnight at −20°C. The fixed cells were washed with PBS and treated with 0.2% (v/v) Triton X-100 (Sigma), RNase A (10 μg/mL) (TaKaRa) and stained with PI (50 μg/mL) for 15 min in the dark. The stained cells were analyzed by using flow cytometry. The cell-cycle assays were performed on a BD Accuri C6 Flow Cytometer (BD Biosciences, United States), and the cell populations at the G0/G1, S, and G2/M phases were quantified using the FlowJo7.6 software.

### Oil Red O Staining and Quantification

The effect of *Dnmt3a1* overexpression and knockdown on the early differentiating preadipocytes was evaluated using an oil red O staining. The oil red O staining and quantification were conducted based on a standard protocol as follows. Cells were washed with PBS and fixed in 4% formaldehyde for 10 min. Subsequently, cells were stained with oil red O working solution [6:4, 0.6% oil red O dye in isopropanol: double distilled water (ddH2O)] for 30 min at room temperature and washed three times with ddH_2_O. The nuclear stain 4′, 6-diamidino-2-phenylindole (DAPI) (Gen-View Scientific Inc., China) was then added and incubated for another 5 min. After the washing process, the number of DAPI-stained cells and lipid droplet accumulation was determined by using a fluorescence inverted light microscope (Leica DMi8, Germany). Finally, oil red O dyes were extracted from the cells in isopropanol solution containing 4% Nonidet P-40 and quantified by a NanoDrop 2000C Spectrophotometers (Thermo Fisher Scientific) at a wavelength of 510 nm.

### Statistical Analysis

Statistical analyses were performed using Microsoft Excel 2010 and the SAS system for Windows v8 software. Statistically significant differences were calculated using Student’s *t*-test. Results are shown as mean ± SEM (standard error of the mean) and differences were considered to be statistically significant when the *P*-value < 0.05.

## Results

### Cloning and Identification of *Dnmt3a* Variant Transcripts in Chicken

Using the 5′-Race method, a novel transcript’s 5′ UTR of chicken *Dnmt3*a was detected (**Supplementary Figure S1**). However, the sequence comparison between the *Dnmt3a* 5′-Race sequence result [817-base pair (bp)] and *Dnmt3a* 5′-UTR (GenBank accession No. NM_001024832.1) is presented in **Supplementary Figure S2**. Using the 3′-Race system, we identified 3′ UTR for *Dnmt3a* (**Supplementary Figure S1**). The 3′-UTR sequence result showed a similar sequence to the one deposited in GenBank accession No: NM_001024832.1. Sequence analysis performed by using the BLAT Search Genome^2^ showed that the chicken *Dnmt3a* (NM_001024832.1.) gene was 13,521 bp long, located on chromosome 3 and spanned from 104,239,221 to 104,252,733 [UCSC Genome Browser on Chicken Nov. 2011 (ICGSC Gallus_gallus-4.0/galGal4) Assembly], and comprised 20 exons.

**FIGURE 1:**
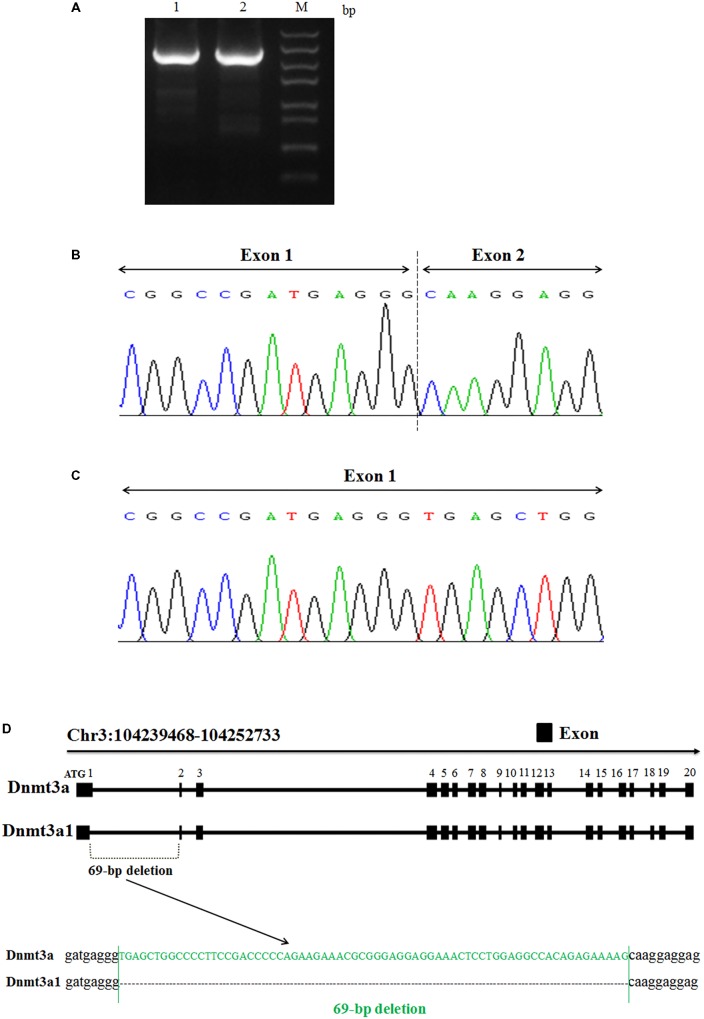
Identification and cloning of a novel *Dnmt3a* splice variant in chicken. **(A)** PCR amplification analysis of two variant transcripts of chicken *Dnmt3a*. The total RNA was extracted from chicken abdominal fat tissue (3-week-old), reverse-transcribed into cDNA and amplified by PCR using the primer pair presented in **Table 1**. The 1.5% agarose gel electrophoresis image of the amplified full-length CDs fragment is shown. M, Trans5k Marker; bp, base pair; 1: full-length CDs product (2634 bp) known as *Dnmt3a*; 2: full-length CDs product (2565 bp) named as *Dnmt3a1*. The amplified products were subjected to sequencing analysis that displays the *Dnmt3a* variant site. **(B)** Variant 1 with 69-bp deletion at the exon-1/exon-2 border (68-bp deleted from exon-1 and 1-bp deleted from exon-2); named *Dnmt3a1*. **(C)** Variant 2 without deletion; named *Dnmt3a*. Both Variant 1 and Variant 2 are results from cDNA sequencing. **(D)** Structure of the two chicken variant transcripts (*Dnmt3a* and *Dnmt3a1*) of *Dnmt3a* is shown. Exons are described by closed squares. The deleted sequences (69-bp) in *Dnmt3a1* are shown in green. The method of detecting the *Dnmt3a* variant transcript is described in Materials and Methods. Chr3:104239468-104252733 is the coding sequence location of *Dnmt3a* (DDBJ accession No: LC381635) and *Dnmt3a1* (DDBJ accession No: LC379990) on chicken chromosome 3 predicted by using the BLAT Search Genome analysis (http://genome.ucsc.edu/cgi-bin/hgBlat) UCSC Genome Browser on Chicken Nov. 2011 (ICGSC Gallus_gallus-4.0/galGal4) Assembly.

The total RNA from chicken abdominal fat tissue was used to clone and sequence the chicken *Dnmt3a* variant transcript. From the sequencing analyses, we identified two variant transcripts for the chicken *Dnmt3a* gene, named *Dnmt3a1* and *Dnmt3a* (**Figures 1A–D**). The complete coding sequences (CDs) of *Dnmt3a1* and *Dnmt3a* have been deposited into the DNA Data Bank of Japan (DDBJ) with accession number LC379990 and LC381635, respectively. The main difference between *Dnmt3a* and *Dnmt3a1* is that *Dnmt3a1* has a deletion of 69-bp encoding 23 amino acids (aa) at the exon-1/exon-2 border (**Figures 1B–D**); 68-bp deleted from exon-1 and 1-bp deleted from exon-2. The amino acid sequence of the Dnmt3a and Dnmt3a1 protein is shown in **Supplementary Figure S3**. In addition, the full-length coding sequence of *Dnmt3a* (DDBJ accession No: LC381635) was similar to the *Dnmt3a* (GenBank accession No: NM_001024832.1); data not shown.

To further investigate the existence of the 69-bp deletion of *Dnmt3a1* in the genomic DNA level, the genomic DNA extracted from the abdominal fat tissue of 3-week-old female Guangxi three-yellow chickens was used as a template for PCR analysis. The result showed that deletion of the 69-bp in *Dnmt3a1* does not exist in the genomic (DNA) level (**Supplementary Figure S7**).

### Expression of *Dnmt3a1* in Adult XH Chicken Tissues and During the Time Course of Adipogenesis

**FIGURE 2:**
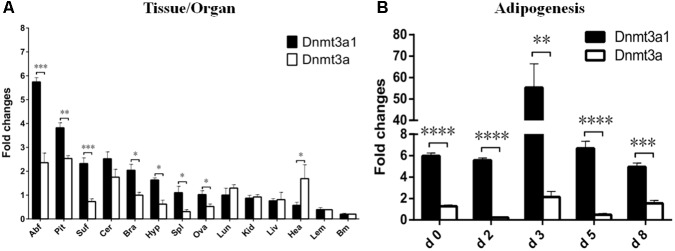
Expression of *Dnmt3a1* in adult XH chicken tissues and during time course of adipogenesis. **(A)** qRT-PCR was performed to examine the mRNA level of the *Dnmt3a1* in female XH chickens at 21 weeks of age. The horizontal axis and vertical axis represent various tissues and their relative mRNA level values. Abf, abdominal fat; Pit, pituitary; Suf, subcutaneous fat; Cer, cerebellum; Bra, brain; Hyp, hypothalamus; Spl, spleen; Ova, ovary; Lun, lung; Kid, kidney; Liv, liver; Hea, heart; Lem, leg muscle; and Brm, breast muscle. Values represent mean ± SEM from three separate experiments (*n* = 6). **(B)** Preadipocytes were induced to differentiate for 8 days. Total RNAs were harvested from the cells at the indicated time points. Day 0 is defined as a day before starting differentiation induction. The method of preadipocyte differentiation is described in Materials and Methods. The level of each mRNA was quantified by qRT-PCR. Data are presented as mean ± SEM from three independent experiments (*n* = 6). ^∗^*P* < 0.05, ^∗∗^*P* < 0.01, ^∗∗∗^*P* < 0.001, and ^∗∗∗∗^*P* < 0.0001.

The relative *Dnmt3a1* mRNA level in various tissues from female XH chicken (at 21 weeks) was detected by qRT-PCR and the quantified data are shown in **Figure 2A**. Expressions were compared between *Dnmt3a1* and *Dnmt3a*. qRT-PCR primers at the specific locations used to differentiate the expression of *Dnmt3a* and *Dnmt3a1* are shown in **Supplementary Figure S5**. Chicken *Dnmt3a1* was ubiquitously expressed in all the 14 tissues examined, and there were significant differences between *Dnmt3a1* and *Dnmt3a* mRNA levels in abdominal fat, pituitary, subcutaneous fat, brain, hypothalamus, spleen, ovary, and heart tissues. The *Dnmt3a1* mRNA level was the highest (2–5.7-fold change) in abdominal fat, pituitary, subcutaneous fat, cerebellum, and brain, and a medium *Dnmt3a1* mRNA level (1–1.6-fold change) was detected in hypothalamus, spleen, ovary, and lung. However, a low (less than onefold change) expression level was observed in other tissues, especially in heart, leg muscle, and breast muscle.

The mRNA level of *Dnmt3a1* was also detected during the time course of adipogenesis. Preadipocytes were isolated from 3-week-old female Guangxi three-yellow chickens. These cells were differentiated *in vitro* for 8 days, and the mRNA level of *Dnmt3a1* was measured before (day 0), during (day 2, 3, and 5), and after differentiation induction (day 8). These results indicated that the mRNA level of both *Dnmt3a1* and *Dnmt3a* peaked on day 3 during differentiation induction, but was significantly higher in *Dnmt3a1* than *Dnmt3a* (**Figure 2B**). Taken together, these data demonstrate that *Dnmt3a1* is highly correlated with adiposity and may function in adipose tissue development.

### Effect of Aging on Tissue-Specific *Dnmt3a1* Expression

**FIGURE 3:**
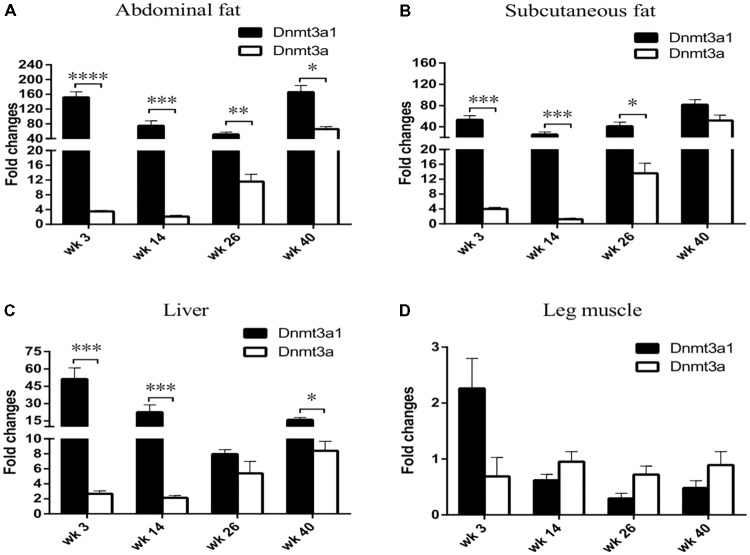
Effects of aging on tissue-specific *Dnmt3a1* expression. *Dnmt3a1* mRNA levels were determined in chicken abdominal fat **(A)**, subcutaneous fat **(B)**, liver **(C)**, and leg muscle **(D)**. To investigate the effect of aging on *Dnmt3a1* mRNA expression, tissues from 3-, 14-, 26-, and 40-week-old female Guangxi three-yellow chickens (*n* = 6/age group) were collected for total RNA isolation. The total RNA was reverse-transcribed into cDNA. The resulting cDNAs were amplified by qRT-PCR with the use of SYBR green as the dye to quantify chicken *Dnmt3a1* mRNA levels. Other details of qRT-PCR assays are reported in Materials and Methods. Data are presented as mean ± SEM from three separate experiments. ^∗^*P* < 0.05, ^∗∗^*P* < 0.01, ^∗∗∗^*P* < 0.001, and ^∗∗∗∗^*P* < 0.0001.

The *Dnmt3a1* transcript was detected in the abdominal fat tissue of 3-week-old chicken. To investigate whether *Dnmt3a1* expression may exist in other chicken ages and in different tissues, its expressions at 3-, 14-, 26-, and 40-week-old chickens were evaluated by qRT-PCR. mRNA levels were compared between *Dnmt3a1* and *Dnmt3a*. Total RNAs were isolated from the abdominal fat, subcutaneous fat, liver, and leg muscle of 3-, 14-, 26-, and 40-week-old chickens, representing important time points in chicken age for producers. Surprisingly, *Dnmt3a1* mRNA levels were strongly expressed in abdominal fat, subcutaneous fat, and liver when compared with *Dnmt3a* mRNA level in all ages evaluated at 3-, 14-, 26-, and 40-week-old chickens (**Figures 3A–C**). The *Dnmt3a1* mRNA level was significantly higher than the *Dnmt3a* mRNA level at 3- and 14-week-old chickens in abdominal fat, subcutaneous fat, and liver. Besides, the *Dnmt3a1* mRNA level was significantly higher than the *Dnmt3a* mRNA level at 26-week-old chickens in abdominal fat and subcutaneous fat, and at 40-week-old chickens in abdominal fat and liver. *Dnmt3a* mRNA expression is upregulated at 26- and 40-week-old chickens compared to 3- and 14-week-old chickens in abdominal fat, subcutaneous fat, and liver. In addition, the *Dnmt3a1* mRNA level was significantly higher in adipose tissue (abdominal fat and subcutaneous fat, 25–165-fold changes; **Figures 3A,B**) compared to non-adipose tissue (leg muscle, 0.3–2.2-fold changes; **Figure 3D**) in all ages examined. These results indicate that the *Dnmt3a1* transcript plays a functional role in adipocyte development compared to muscle development. Chicken *Dnmt3a1* and *Dnmt3a* expressions were regulated by aging.

### Effect of Fasting or a High-Fat Diet on Tissue-Specific *Dnmt3a1* Expression

**FIGURE 4:**
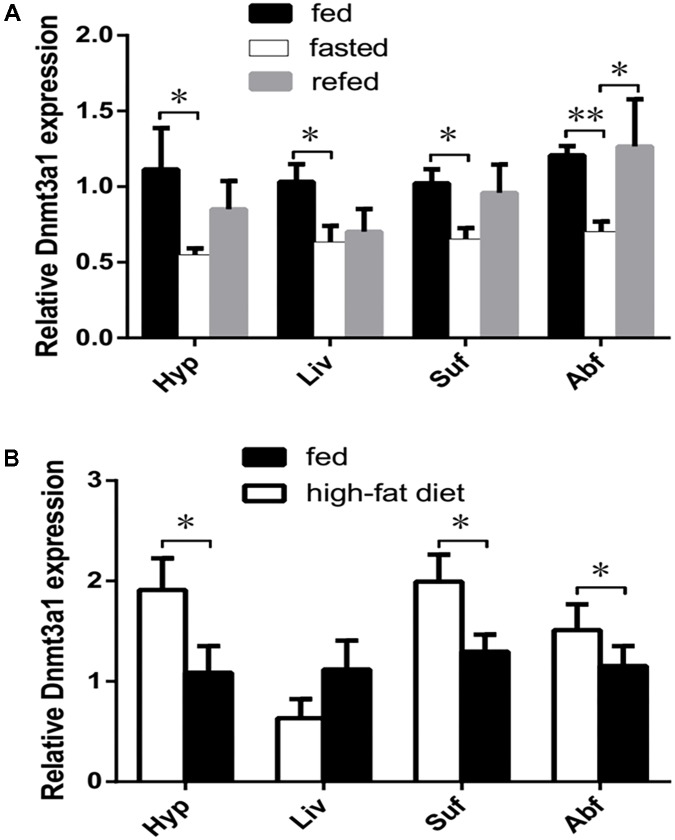
Effect of different feeding statuses on tissue-specific *Dnmt3a1* expression. **(A)** *Dnmt3a1* mRNA levels in chow-fed, fasted, and refed female XH chicken at 24 weeks of age. Tissues (Hyp, Liv, Suf, and Abf) from fed, fasted (for 3 days), and refed chickens for 1 day (*n* = 6/case) were harvested for the quantification of *Dnmt3a1* mRNA by qRT-PCR as presented in Materials and Methods. Values represent mean ± SEM from three independent experiments. ^∗^*P* < 0.05 and ^∗∗^*P* < 0.01. **(B)** The effect of a high-fat diet vs. a standard diet on the *Dnmt3a1* mRNA level in female XH chicken at 24 weeks of age. The chickens were either fed a high-fat diet or a standard diet (chow diet) for 3 weeks. *P*-values are represented with asterisks (^∗^, *P*-value ≤ 0.05), and each bar represents mean ± SEM from three independent experiments (*n* = 6 per group).

The *Dnmt3a1* mRNA level was significantly decreased in response to fasting for 3 days in four tissues (*P* < 0.05; in the hypothalamus, liver, and subcutaneous fat, or *P* < 0.01; in the abdominal fat) examined, whereas refeeding for 24 h increased these levels with a significantly higher level (*P* < 0.05) in abdominal fat compared with the fasting group (**Figure 4A**; *n* = 6 in each group).

Consuming a high-fat diet for 3 weeks significantly increased the *Dnmt3a1* expression in the hypothalamus, subcutaneous fat, and abdominal fat tissues examined (*P* < 0.05). In contrast to these tissues, the *Dnmt3a1* mRNA level in the liver did not differ (*P* > 0.05) between a high-fat diet and a standard chow diet (**Figure 4B**; *n* = 6 in each group). The total body weight gain of the chicken on a high-fat diet was higher (but not significantly; *P* > 0.05) than in the control group (data not shown).

### *Dnmt3a1* Inhibits Preadipocyte Proliferation and Cell-Cycle Distribution

**FIGURE 5:**
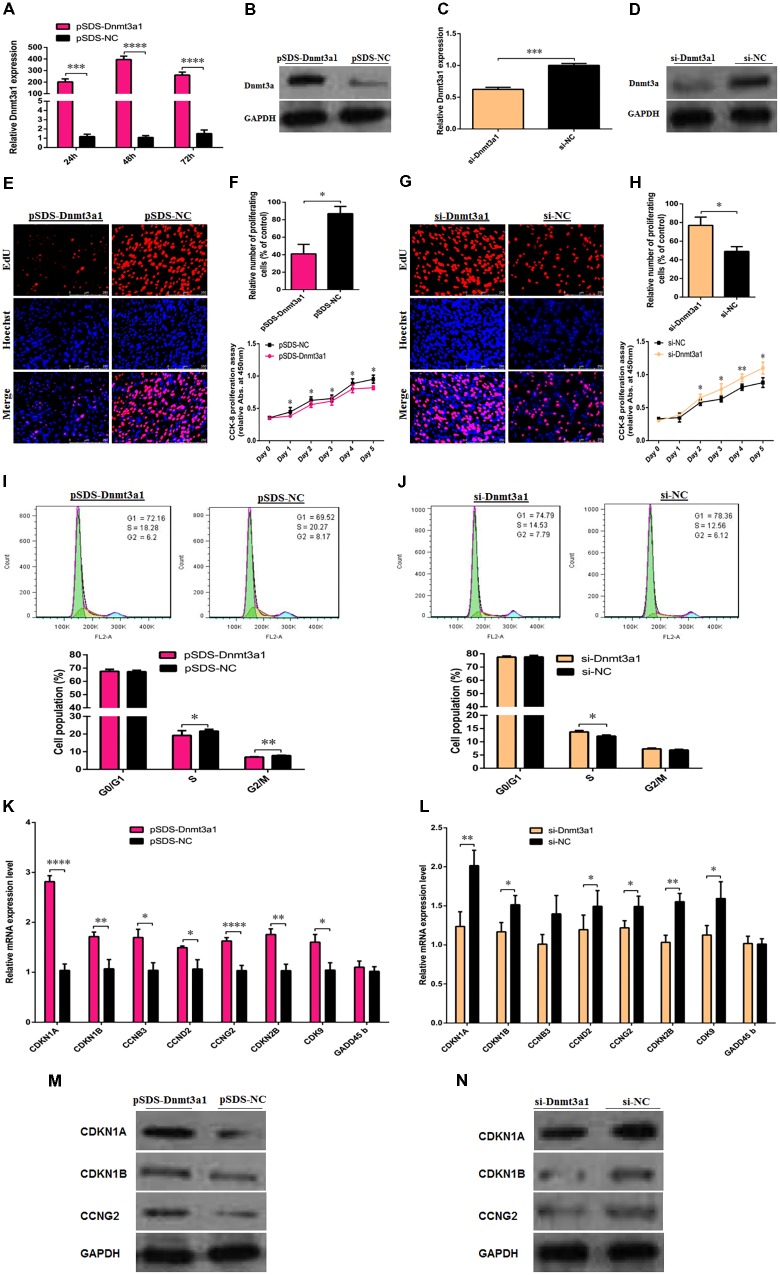
*Dnmt3a1* inhibits preadipocyte proliferation and cell-cycle progression. The efficiency of *Dnmt3a1* overexpression was evaluated in preadipocytes at 24, 48, and 72 h after transfection by qRT-PCR **(A)** or at 48 h after transfection by Western blotting **(B)**. *Dnmt3a1* siRNA efficiency was examined in preadipocytes at 48 h after transfection by qRT-PCR **(C)** or by Western blot analysis **(D)**. In all the above panels, data are expressed as the mean ± SEM of six independent experiments. ^∗∗^*P* < 0.01, ^∗∗∗^*P* < 0.001, and ^∗∗∗∗^*P* < 0.0001. **(E)** Representative images of EdU incorporation assay (red) and DAPI (blue) are presented between preadipocytes overexpressed with pSDS-Dnmt3a1 and preadipocytes subjected to pSDS-NC (controls); cells were transfected at 85% confluence, scale bar: 250 μm. [**(F)**; *upper panel*] Statistical results of the percentage of EdU-positive cells between pSDS-Dnmt3a1 and pSDS-NC. Values represent mean ± SEM from three independent experiments. ^∗^*P* < 0.05. [**(F)**; *lower panel*] Cell proliferation or viability was monitored by CCK-8 assay in pSDS-Dnmt3a1 or pSDS-NC; data are presented as means ± SEM of six independent experiments with ten replicates in each. ^∗^*P* < 0.05. **(G)** Representative images of EdU incorporation assay (red) and DAPI (blue) are showed between preadipocytes treated with si-Dnmt3a1 or preadipocytes treated with si-NC; cells were transfected at 70% confluence, scale bar: 250 μm. [**(H)**; *upper panel*] Statistical results of the percentage of EdU-positive cells between si-Dnmt3a1 and si-NC. Values represent mean ± SEM from three independent experiments. ^∗^*P* < 0.05. [**(H)**; *lower panel*] Cell proliferation was monitored by CCK-8 assay in si-Dnmt3a1 or si-NC; data are presented as means ± SEM of six independent experiments with ten replicates in each. ^∗^*P* < 0.05 and ^∗∗^*P* < 0.01. [**(I)**; *upper panel*] Cell-cycle analysis of proliferating preadipocytes was evaluated by flow cytometry between pSDS-Dnmt3a1 and pSDS-NC. [**(I)**; *lower panel*] Quantitative results of flow cytometry experiments between pSDS-Dnmt3a1 and pSDS-NC in proliferating preadipocytes. Values represent mean ± SEM from three independent experiments. ^∗^*P* < 0.05 and ^∗∗^*P* < 0.01. [**(J)**; *upper panel*] Cell-cycle analysis was determined by flow cytometry between si-Dnmt3a1 and si-NC treated cells. [**(J)**; *lower panel*] Quantitative results of flow cytometry assays between proliferating preadipocytes treated with si-Dnmt3a1 compared with proliferating preadipocytes treated with si-NC. Values represent mean ± SEM from three independent experiments. ^∗^*P* < 0.05. **(K)** Relative mRNA levels of cell-cycle-related genes after transfection with pSDS-Dnmt3a1 and pSDS-NC in proliferating preadipocytes, determined by qRT-PCR at 48 h post-transfection. Values represent mean ± SEM from four independent experiments. ^∗^*P* < 0.05, ^∗∗^*P* < 0.01, and ^∗∗∗∗^*P* < 0.0001. **(L)** Relative mRNA levels of cell-cycle-related genes after transfection with si-Dnmt3a1 and si-NC in proliferating preadipocytes, determined by qRT-PCR at 48 h post-transfection. Values represent mean ± SEM from four independent experiments. ^∗^*P* < 0.05 and ^∗∗^*P* < 0.01. **(M)** Protein expressions of cell-cycle-related genes were detected by Western blot analysis after transfection with pSDS-Dnmt3a1 and pSDS-NC in proliferating preadipocytes; band intensity ± SEM from three independent experiments. **(N)** Protein expressions of cell-cycle-related genes were detected by Western blot analysis after transfection with si-Dnmt3a1 and si-NC in proliferating preadipocytes. Band intensity of Western blotting was obtained by averaging the data from three separate experiments.

To determine the potential role of *Dnmt3a1* in chicken primary preadipocyte proliferation and cell-cycle progression, *Dnmt3a1* overexpression (pSDS-Dnmt3a1 vs. pSDS-NC) and knockdown (si-Dnmt3a1 vs. si-NC) experiments were performed. The overexpression efficiency of *Dnmt3a1* was confirmed by qRT-PCR, Western blot analysis, and global 5-methylcytosine analysis. As shown in **Figures 5A,B** and **Supplementary Figure S4**, Dnmt3a1 was effectively overexpressed. In addition, after a series of siRNA transfection experiments, an optimum concentration of *Dnmt3a1* siRNA was achieved at 50 nM (data not shown). si-Dnmt3a1 that specifically targeted chicken *Dnmt3a1* mRNA is shown in **Supplementary Figure S6**. Dnmt3a1 knockdown efficiency (using siRNA targeting *Dnmt3a1*) was also confirmed by qRT-PCR and Western blot analysis (**Figures 5C,D**).

To elucidate the effects of *Dnmt3a1* on cell proliferation, EdU and CCK-8 assays were performed. The EdU assay showed that the mean percentage of positive proliferative cells of the pSDS-Dnmt3a1 group reduced by 46% (**Figures 5E,F**; *top panel*). Compared with the pSDS-NC group, the cell viability numbers by CCK-8 decreased significantly in the pSDS-Dnmt3a1 group, from day 1 to day 5 after incubation (**Figure 5F**; *bottom panel*). In contrast to pSDS-Dnmt3a1, the mean percentage of the positive proliferative cells of the si-Dnmt3a1 group increased by 28% (**Figures 5G,H**; *top panel*) as judged by the EdU assay. Compared with the si-NC group, the cell numbers significantly increased in the si-Dnmt3a1 group from day 2 to day 5 after incubation (**Figure 5H**; bottom panel).

To further examine the effects of *Dnmt3a1* on preadipocyte proliferation, flow cytometry assays were used to evaluate cell-cycle distribution. The overexpression of *Dnmt3a1* significantly decreased the numbers of S-phase and G2/M-phase cells and the induced cell-cycle arrest (**Figure 5I**). Conversely, *Dnmt3a1* knockdown significantly increased S-phase cells relative to control group cells (**Figure 5J**). Together, these data indicate that *Dnmt3a1* expression has a negative regulatory role on cell proliferation and decreases the cell-cycle distribution of proliferating preadipocytes.

Next, to further identify whether *Dnmt3a1* regulates preadipocyte proliferation through cell-cycle distribution, the relative mRNA level and protein expression of several marker genes for triggering the major cell-cycle transitions were detected after the overexpression or RNA interference of *Dnmt3a1*. The overexpression of *Dnmt3a1* significantly upregulated the mRNA level of cell-cycle-related genes, such as *CDKN1A*, *CDKN1B*, *cyclin B3* (*CCNB3*), *cyclin D2* (*CCND2*), *CCNG2*, *cyclin-dependent kinase inhibitor 2B* (*CDKN2B*), and *CDK9*, and upregulated the protein level of CDKN1A, CDKN1B, and CCNG2 (**Figures 5K,M**). Conversely, the knockdown of *Dnmt3a1* by siRNA had the opposite effect (**Figures 5L,N**). However, the *growth arrest and DNA damage inducible beta* (*GADD45b*) mRNA level was not significantly changed after *Dnmt3a1* overexpression or knockdown (*P* > 0.05). These results suggest that *Dnmt3a1* modulates multiple cell-cycle gene expressions in preadipocyte and then inhibits cell proliferation.

### *Dnmt3a1* Decreases Lipid Droplet Accumulation in the Early Stage of Adipocyte Differentiation

**FIGURE 6:**
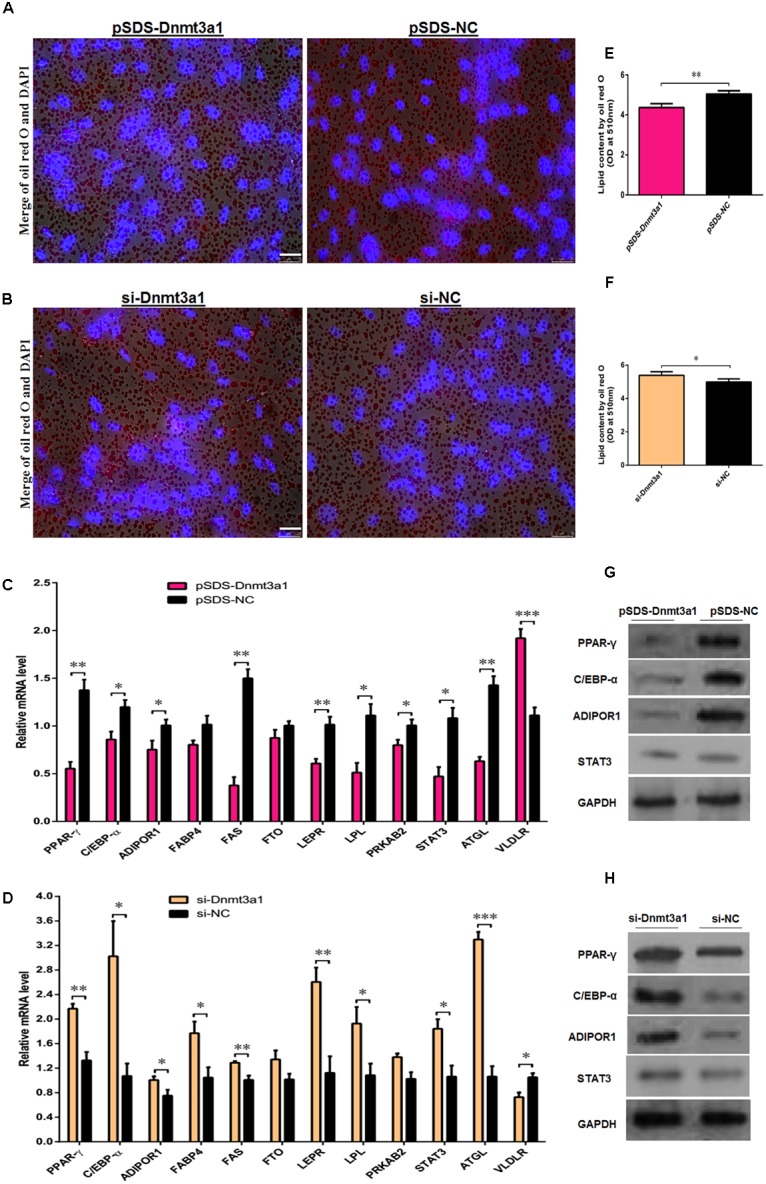
*Dnmt3a1* decreases lipid droplet accumulation in the early stage of adipocyte differentiation. **(A)** Representative images of oil red O staining (red) and DAPI staining (blue) after transfection with pSDS-Dnmt3a1 and pSDS-NC for 48 h are showed; scale bar: 25 μm. **(B)** Lipid droplet content by oil red O staining and extraction method of cells transfected with pSDS-Dnmt3a1 and pSDS-NC for 48 h. Values represent mean ± SEM from three independent experiments. ^∗∗^*P* < 0.01. **(C)** Representative images of oil red O staining (red) and DAPI staining (blue) after transfection with si-Dnmt3a1 and si-NC for 48 h are showed; scale bar: 25 μm. **(D)** Lipid droplet content by oil red O staining and the extraction method of cells transfected with si-Dnmt3a1 and si-NC for 48 h. Values represent mean ± SEM from three independent experiments. ^∗^*P* < 0.05. **(E)** Relative mRNA level of some general genes implicated in adipogenesis or energy homeostasis induced by pSDS-Dnmt3a1 and pSDS-NC into cells. Values represent mean ± SEM from four separate experiments. ^∗^*P* < 0.05, ^∗∗^*P* < 0.01, and ^∗∗∗^*P* < 0.001. **(F)** Protein expression of some general adipose genes induced by pSDS-Dnmt3a1 and pSDS-NC into cells. **(G)** Relative mRNA level of some general genes involved in adipogenesis or energy homeostasis after transfection with si-Dnmt3a1 and si-NC into cells for 48 h. Values represent mean ± SEM from four separate experiments. ^∗^*P* < 0.05, ^∗∗^*P* < 0.01, and ^∗∗∗^*P* < 0.001. **(H)** Protein expression of some general genes involved in adipogenesis at 48 h after transfection by si-Dnmt3a1 and si-NC into cells. The band intensity of Western blotting was obtained by averaging the data from three independent experiments.

We investigated whether the overexpression or knockdown of *Dnmt3a1* had any effect on lipid droplet formation during the differentiation of preadipocytes to adipocytes. These cells were (*in vitro*) induced to differentiate for 8 days. First, *Dnmt3a1* mRNA levels were analyzed before and after starting the differentiation process; these results are shown in **Figure 2B**. As we mentioned earlier, *Dnmt3a1* mRNA levels were upregulated during the time course of differentiation. Because we did not have or produce the antibody for this novel transcript *Dnmt3a1*, we were not able to perform Dnmt3a1 protein expression analysis. Differentiation of preadipocytes to adipocytes was completed within 7 days, and this was confirmed by oil red O staining assays (exogenous and endogenous lipid formation). On day 2 of preadipocyte differentiation induction, we transfected these cells using transient overexpression or knockdown of *Dnmt3a1* for 48 h, and then the lipid droplet accumulations were analyzed. As judged by oil red O staining and extraction assays, the overexpression of *Dnmt3a1* significantly decreased lipid droplet formation in the early stage of differentiation (**Figures 6A,B**). Conversely, *Dnmt3a1* knockdown significantly increased lipid droplets relative to control (**Figures 6C,D**).

Next, to further investigate the role of *Dnmt3a1* in the adipogenesis, the mRNA level of genes in relation to adipose or glucogenesis, such as *PPAR-γ, C/EBP-α, adiponectin receptor 1* (*ADIPOR1*), *fatty acid-binding protein 4* (*FABP4*), *fatty acid synthase* (*FAS*), *fat mass and obesity-associated* (*FTO*), *leptin receptor* (*LEPR*), *lipoprotein lipase* (*LPL*), *protein kinase AMP-activated non-catalytic subunit beta 2* (*PRKAB2*), *signal transducer and activator of transcription 3* (*STAT3*), *patatin-like phospholipase domain-containing 2* (*ATGL*), and *very-low-density lipoprotein receptor* (*VLDLR*) were detected by qRT-PCR after the overexpression or knockdown of *Dnmt3a1*. In addition, the protein level of PPAR-γ, C/EBP-α, ADIPOR1, and STAT3 were also detected by Western blot analysis. The differentiating adipocytes were harvested at 48 h after transfection and subjected to either RNA extraction or cellular protein extraction for further expression analysis. As shown in **Figures 6E,F**, the overexpression of *Dnmt3a1* significantly decreased the mRNA and protein expression of PPAR-γ, C/EBP-α, ADIPOR1, and STAT3, and also the mRNA expression of *FAS, LEPR, LPL, PRKAB2*, and *ATGL.* In contrast, their expression was significantly increased after RNA interference with *Dnmt3a1* (**Figures 6G,H**). However, the overexpression or knockdown of *Dnmt3a1* significantly increased or decreased, respectively, the mRNA levels of *VLDLR* (**Figures 6E,G**). Taken together, these data suggest that *Dnmt3a1* may also control the early stage of adipocyte differentiation.

## Discussion

*Dnmts* (*Dnmt1*, *Dnmt2*, *Dnmt3a*, *Dnmt3b*, and *Dnmt3L*) are the enzymes that methylate cytosine in the 5-position of cytosine bases, mostly within CpG dinucleotides, and the corresponding genes are clearly different for their various roles (Cheng and Blumenthal, 2008). *Dnmt1* enhances DNA methylation after DNA replication and plays a major role in the maintenance of DNA methylation, while the *Dnmt3* family is essential for generating the *de novo* methylation pattern during embryogenesis and development (Okano et al., 1999; Chen and Li, 2006). *Dnmt3a* knockout mice exhibit different defects and die at different developmental stages (Okano et al., 1999). A recent study shows that the *Dnmts* [*Dnmt1*, *Dnmt1-associated protein 1* (*Dmap1*), *Dnmt3a*, *and Dnmt3b*] are tissue-specifically responsive to acute and chronic stress in chickens (Kang et al., 2017). The chicken *Dnmt3a* gene is located on chromosome 3 and contains an open reading frame of 2634-bp, which encodes 877 amino acids (GenBank accession No: NM_001024832.1). Furthermore, the chicken Dnmt3a protein had two completely distinct functional domains, namely the PWWP domain (at site 254–327 aa, *e*-value 1.4e–23) and the domain of C-5 cytosine-specific DNA methylase (at site 599–741 aa, *e*-value 1.7e–12), which are conserved across vertebrate species (Rengaraj et al., 2011). As compared to the novel transcript of chicken *Dnmt3a*, *Dnmt3a1* does not lack either the conserved PWWP domain or the C-5 cytosine-specific DNA methylase domain.

In humans or mice, several alternatively spliced variants of *Dnmts* have been characterized in normal or cancer cells (Mertineit et al., 1998; Okano et al., 1998; Robertson et al., 1999; Saito et al., 2002; Weisenberger et al., 2002). Using Northern blot analysis, two variant transcripts of *Dnmt3a* with different lengths have been detected in mouse 4.0 kb, 4.2 kb; in length and humans 4.4 kb and 9.5 kb; in length (Okano et al., 1998; Robertson et al., 1999; Xie et al., 1999). In humans and mice, a shorter isoform of *Dnmt3a2* was identified (Chen et al., 2002). The human Dnmt3a2 protein lacks 223 aa, while the mouse Dnmt3a2 protein lacks 219 aa at the N-terminal residues of the full-length Dnmt3a. However, the exact function of the variant *Dnmt3a* transcripts is largely unknown (Okano et al., 1998). Herein we report the identification of two *Dnmt3a* (coding sequence) transcript variants; one is a novel transcript (alternate splicing) of chicken *Dnmt3a* named *Dnmt3a1* that differs from *Dnmt3a* by lacking 69-bp (encoding 23 amino acids) at the exon-1/exon-2 border. Deletion of the 69-bp in *Dnmt3a1* does not exist in the genomic (DNA) level (**Supplementary Figure S7**). To our knowledge, no study has identified deletion in the coding sequence across the exon-1/exon-2 boundary of *Dnmt3a* mRNA in avians or mammals. In chicken, deletions in the part of the exon or three-exon deletions with another deletion at the exon/exon border in the coding sequences of *FTO* variant transcripts have been reported (Jia et al., 2012). Unfortunately, Jia et al. (2012) did not include in their report whether the sequence deletion exists in the genomic DNA level. The 69-bp deletion in *Dnmt3a1 mRNA* does not change the reading frame of base-pair triplets (**Supplementary Figure S3**). We think that *Dnmt3a* and *Dnmt3a1* are generated from the same genomic DNA sequence, and the 69-bp deletion is caused by alternative splicing. It has been reviewed that when two or more splice sites compete, alternative splicing generates mRNA variant transcripts that produce different amino acids from a single gene (Black, 2003). Interestingly, the overexpression of *Dnmt3b4* in human epithelial 293 cells induced DNA demethylation (Saito et al., 2002). However, in this study, the percentage of global DNA methylation was significantly increased after *Dnmt3a1* overexpression in proliferating preadipocytes. This result explains that the endogenous function of DNA methyltransferase activity exists with the *Dnmt3a1* transcript, or there might be another regulatory mechanism for these two transcripts (*Dnmt3a* and *Dnmt3a1*) that requires further investigation.

In the present study, *Dnmt3a1* is ubiquitously expressed in various tissues of female chickens with the highest level in adipose tissues (abdominal fat and subcutaneous fat) and pituitary, and it also highly expressed in primary preadipocytes and during preadipocyte differentiation. Furthermore, the *Dnmt3a1* mRNA level is highly expressed in adipose tissue and liver during aging. Studies have reported that adipocytes are a major source of free fatty acid synthesis in some mammals, such as dog and cat, while liver is largely considered as a major source of *de novo* lipogenesis in avians and humans (Leveille et al., 1975; Patel et al., 1975; Stangassinger et al., 1986; Richard et al., 1989). However, according to Griffin et al. (1992) and Resnyk et al. (2015), abdominal adipocytes could make a remarkable contribution to fatty acid synthesis in avians than previously thought. In chicken, *Dnmt3a* is strongly expressed during early embryonic development, but it is expressed with moderate levels in all somatic and gonadal samples of male and female embryos. Additionally, *Dnmt3a* mRNA levels in female and male gonads were observed to be at a low level from embryonic day 8.5 to embryonic day 14.5, and then were maintained at a basal level until 25 weeks as detected in the testes and ovaries of chickens (Rengaraj et al., 2011). *Dnmt3a* is widely expressed at low levels and localizes to heterochromatin, revealing a housekeeping function. In contrast, *Dnmt3a2* has been proposed to be more essential for *de novo* methylation since it expresses at high levels in embryonic stem cells and displays restricted expression in tissues known to undergo *de novo* methylation, such as the ovaries and testes (Okano et al., 1998; Chen et al., 2002; Weisenberger et al., 2004). Human *Dnmt3a* is expressed in a variety of tissues (Robertson et al., 1999; Xie et al., 1999).

In the current study, the expression patterns of *Dnmt3a1* were investigated in different tissues including abdominal fat, subcutaneous fat, liver, and leg muscle in four distinct developmental stages in chickens (3-, 14-, 26-, and 40-week-old), representing important time points of growth rate in the life of chickens for farmers. Interestingly, the expression of *Dnmt3a1* was seen to be regulated by aging, since 3-week-old female chicks expressed higher mRNA levels than that of 14- and 26-week-old chickens (gradually declined), especially in abdominal fat, liver, and leg muscle, and then the *Dnmt3a1* expression started to increase in 40-week-old chickens in almost all tissues examined. In mice, *Dnmt3a* mRNA levels significantly decreased during aging in the frontal cortex and the cerebellum of the brain (Kraus et al., 2016). Because chicken *Dnmt3a1* and *Dnmt3a* transcripts show a significant difference in expression patterns during aging, they may have distinct functions in stages of aging development. However, their exact mechanisms and functions in aging development may require further investigation. The high impact of fasting and high-fat diets on *Dnmt3a1* expression strongly suggests a role of *Dnmt3a1* in energy and fat metabolism. Under different feeding regimes, *Dnmt3a1* expression was downregulated under fasting conditions in four tissues examined, including hypothalamus, liver, subcutaneous fat, and abdominal fat. The high-fat diet increased the *Dnmt3a1* expression in the hypothalamus, subcutaneous fat, and abdominal fat of chicken. A previous study showed that the high-fat diet increased the *Dnmt3a* expression in the adipose tissue of obese mice (Kamei et al., 2010). In this study, a high-fat diet (25% fat) had a greater total body weight gain than control diet (5% fat) after 3 weeks of feeding in XH chickens. In a previous study in which male broilers were fed diets formulated with the same protein and metabolizable energy contents but differing in having either high-fat, high-fiber contents (HF) or low-fat, low-fiber contents (LF), the growth performance of genetically fat or lean chickens did not significantly change. However, the percentage of polyunsaturated fatty acids was lower for the LF diet than for the HF diet. The enzymatic activity of FAS was significantly affected by diet treatments in the lean or fat chickens, and was lower in the HF diet (Baeza et al., 2015). By contrast, chickens fed a high-fat diet and low protein showed higher abdominal fat deposition than chickens fed a low-fat and high-protein diet, and this difference was more remarkable for fat chickens (Swennen et al., 2006). These discrepancies are probably due to the differences in the degree of substitution of energy values, but do not detract from the main finding of the present study.

Adipogenesis is a process regulated by a highly constructed network with many transcriptional factors including PPAR-γ and C/EBP family proteins (Gregoire et al., 1998; Lefterova et al., 2008; Madsen et al., 2014). During adipogenesis, preadipocytes undergo cell-cycle arrest followed by the reconstitution of gene expression programs, leading to the accumulation of lipid and adipocyte phenotypes (Brun et al., 1996; Aguilar et al., 2010; Lefterova et al., 2014). These alterations in gene transcription are tightly coordinated with alterations in the cell cycle. Cell-cycle progression is controlled by the equilibration between growth-inducing and growth-inhibitory signals that concur through prominent signaling networks on the cell-cycle machinery. This cell-cycle regulatory machinery consists of cyclins, cyclin-dependent kinases (CDKs), and their inhibitors, cyclin-dependent kinase inhibitors (CKIs). These proteins cooperate to control the cell-cycle distribution at particular regulatory points of the cell cycle by directing the cell toward a response of proliferation, growth arrest, or apoptosis (Hartwell and Kastan, 1994). All the CIP/KIP family proteins contain distinct cyclin and CDK-binding domains near the N-terminus, and the interaction of these proteins with cyclin/CDK complexes is enough to compel the cell-cycle arrest during the G1/S phase of the cell cycle. *CKI* belongs to the CIP/KIP family, which also includes *p21* ^(CIP1/WAF1)^ and *p27* ^(KIP1)^ (Besson et al., 2008). Previous studies have demonstrated that cell-cycle-related genes or proteins, such as CDKN1B/p21, CDKN1B/P27, CCNG2, and CDK9 are implicated in adipogenesis *in vitro* or *in vivo* (Lin et al., 2003; Nam et al., 2008; Aguilar et al., 2010). Many approaches have been proposed to study the function of transcripts and some had already provided evidence for the importance of studying transcripts (Okano et al., 1998; Siewiera et al., 2015; Ahmad et al., 2017). To evaluate the biological function of the *Dnmt3a1* transcript, we performed *in vitro* transient overexpression and knockdown of *Dnmt3a1* in proliferating preadipocytes and during the early differentiating adipocytes. Our EdU, CCK-8, and flow cytometry methods indicate that *Dnmt3a1* significantly inhibited the proliferation of primary preadipocytes. Additionally, during preadipocyte proliferation, we found that the overexpression of *Dnmt3a1* significantly upregulated the mRNA and protein expression of cell-cycle-related genes (CDKN1A, CDKN1B, and CCNG2), whereas the knockdown of *Dnmt3a1* by siRNA had the opposite effects. Previous studies have shown that the overexpression of *CDKN1A* could inhibit the cell-cycle progression (El-Deiry et al., 1993; Zakut and Givol, 1995). *CDKN1A* has the capability to bind to the *CDK* holoenzyme and inhibit *CDK* complexes that are necessary for G1 progression and S-phase entry. It could be targeted by a transcriptional factor complex and inhibit fat-cell differentiation (Nakae et al., 2003). The enhanced protein expression of p27 results in cellular arrest by binding to cyclin/CDK complexes. In adult mice overexpressing *CDKN1B*, the cell proliferation level was decreased in all tissues examined (brain-subventricular zone, skeletal muscle, skin, and intestine) (Pruitt et al., 2013). It was previously indicated that the expression of *CDKN1A* and *CDKN1B* is altered during adipogenesis (Morrison and Farmer, 1999). *CCNG2* and its closest homolog *cyclin G1* have been identified as a unique family of *cyclins* (Horne et al., 1996). *CCNG2* is a key negative regulator of cell-cycle distribution in cells (Bennin et al., 2002; Ahmed et al., 2012). CCNG2 has been found to be directly interacting with protein phosphatase 2 catalytic subunit alpha (PPP2CA) to inhibit cell proliferation (Bennin et al., 2002). *CCNG2* expression is upregulated by the activation of *forkhead box O1* (*FoxO1*) and *FoxO3* (Martinez-Gac et al., 2004). *Dnmt3a1* overexpression upregulates the mRNA level of *CCNB3*, *CCND2*, *CDKN2B*, and *CDK9* in proliferating preadipocytes, whereas the knockdown of *Dnmt3a1* has opposite effects. Together, these data suggest that *Dnmt3a1* might coordinately regulate preadipocyte proliferation by upregulating *CDKN1A*, *CDKN1B*, and *CCNG2* expression.

In this study, *Dnmt3a1* overexpression or knockdown during early adipocyte differentiation altered the expression of some common genes, which were previously reported to play important roles in adipogenesis. For example, both *PPAR-γ* and *C/EBPs* are critical regulators for adipogenesis (Hartwell and Kastan, 1994; Brun et al., 1996; Gregoire et al., 1998; Lefterova et al., 2014; Madsen et al., 2014; Kraus et al., 2016; Abdalla et al., 2018). *ADIPOR*1 acts as the main receptor for adiponectin and has essential roles in the regulation of lipid metabolism and energy homeostasis (Yamauchi et al., 2007). *FAP4* is active in both adipogenic precursors and mature adipocytes (Shan et al., 2013). *STAT3* promotes adipogenesis (Yuan et al., 2017). *FAS*, *LEPR*, *LPL*, *PRKAB2*, and *ATGL* have been previously reported to be involved in fat metabolism or energy homeostasis (Hermier et al., 1989; Ailhaud et al., 1992; Gregoire et al., 1998; Kershaw and Flier, 2004; Zimmermann et al., 2004; Dasgupta et al., 2012). In this study, the mRNA and protein expression of PPAR-γ, C/EBP-α, ADIPOR1, and STAT3, and the mRNA levels of *FAS*, *LEPR*, *LPL*, *PRKAB2*, and *ATGL* in early differentiating adipocytes were significantly affected because of either overexpression or knockdown of *Dnmt3a1*. *Dnmt3a1* overexpression or knockdown also decreased or increased, respectively, the expression of *FTO*, but this effect is less obvious and is not significant. *VLDL* transfers endogenous triglycerides, phospholipids, and cholesterol to peripheral tissues. It acts as the body’s internal export mechanism for fats probably through its receptor (*VLDLR*). *VLDL* (with apoprotein B-100, apo B) is synthesized in the liver except that the triglyceride-carrying occurs in the white adipocyte tissues (Queiroz et al., 2010). The upregulation of *VLDLR* expression by *Dnmt3a1* during early adipocyte differentiation indicates that *Dnmt3a1* might regulate fat balance through *VLDLR*. In this study, the effects of *Dnmt3a1* overexpression and knockdown on lipid droplet accumulations were evaluated. The overexpression of *Dnmt3a1* significantly decreased lipid droplet accumulation in the early stage of differentiating adipocytes, while the knockdown of *Dnmt3a1* had the opposite effects. These data strongly suggest that *Dnmt3a1* might coordinately regulate all the stages of adipogenesis in chicken.

Although the intensive selection in chickens has increased daily growth rate and body weight, and reduced market age, commercial broilers have an increased tendency toward physiological disorders such as obesity (Li et al., 2003). Results of this study explain that chickens, particularly commercial broilers, can be used as a research model to study obesity or obesity-related diseases, as far as this unique *Dnmt3a1* transcript was detected in the chicken abdominal fat. Studying the molecular basis of adipogenesis and adipocyte function in chicken leads to expand our knowledge to the non-mammalian species. There are fundamental similarities between chicken and human genomes (Hillier et al., 2004). These could help us identify the functional elements and regulatory circuits. For example, the chicken genome is significantly smaller in size than the human genome but with almost the same number of genes. This explains why the chicken genome has a substantial reduction in DNA duplications, DNA repeat sequences, and fewer pseudogenes. About 60% of chicken genes correspond to almost the same as a human gene. The finding that *Dnmt3a1* inhibits preadipocyte proliferation and early adipocyte differentiation has important implications for our understanding of adipogenesis and might help in treating or preventing obesity. However, future experiments may be required to confirm the role of *Dnmt3a1* in fat metabolism through RNA sequencing and proteomics analyses during chicken adipogenesis after *Dnmt3a1* overexpression and knockdown. These databases can be used to demonstrate the effect of *Dnmt3a1* on key genes/proteins related to fat metabolism at the global level. Producing antibody against Dnmt3a1 is necessary to validate Dnmt3a1 target genes and study Dnmt3a1 protein expression.

In conclusion, two variant transcripts were identified for the chicken *Dnmt3a* gene. Variant *Dnmt3a1* is a novel transcript. It is highly expressed in adipose tissue and during the time course of adipogenesis. *Dnmt3a1* expression was affected by aging, fasting, and high-fat diet. Furthermore, *Dnmt3a1* inhibited preadipocyte proliferation and their subsequent differentiation. Our data indicate that *Dnmt3a1* has a potential role in fat metabolism.

## Author Contributions

BA designed the study, performed all the experiments, analyzed the data, and drafted the manuscript. ZL and HO analyzed the data and provided essential logistical help. EJ, TS, and J-aY conducted some experiments and harvested tissue samples. BoC and BiC analyzed the data and provided essential help. QN designed the experimental approach and oversaw the work. XZ participated in the design of the study.

## Conflict of Interest Statement

The authors declare that the research was conducted in the absence of any commercial or financial relationships that could be construed as a potential conflict of interest.

## Footnotes

1 www.sidansai.com

2 http://genome.ucsc.edu/cgi-bin/hgBlat
