# Supplementary material for: A Novel Dnmt3a1 Transcript Inhibits Adipogenesis
Source: Front Physiol. 2018 Oct 2;9:1270. doi: 10.3389/fphys.2018.01270 (PMC6176318; doi:10.3389/fphys.2018.01270)
Supplement: Supplementary file 1 [file Data_Sheet_1.docx]

**A Novel *Dnmt3a1* Transcript Inhibits Adipogenesis**

Bahareldin A. Abdalla ^1,2,3^, Zhenhui Li ^1,2,3^, Hongjia Ouyang ^1,2,3^, Endashaw Jebessa^1,2,3^, Tianhao Sun ^1,2,3^, Jia-ao Yu^1,2,3^, Bolin Cai ^1,2,3^, Biao Chen^1,2,3^, Qinghua Nie ^1,2,3,*^ and Xiquan Zhang ^1,2,3^

*^1^* *Department of Animal Genetics, Breeding and Reproduction, College of Animal Science, South China Agricultural University, Guangzhou 510642, Guangdong, China, ^2^ National-Local Joint Engineering Research Center for Livestock Breeding, ^3^ Guangdong Provincial Key Lab of Agro-Animal Genomics and Molecular Breeding, and the Key Lab of Chicken Genetics, Breeding and Reproduction, Ministry of Agriculture, Guangzhou, China*

***Correspondence:** Qinghua Nie, E-mail: [nqinghua@scau.edu.cn](mailto:nqinghua@scau.edu.cn)

**Running title:** A Novel *Dnmt3a1* Transcript Inhibits Adipogenesis

SUPPLEMENTARY MATERIAL


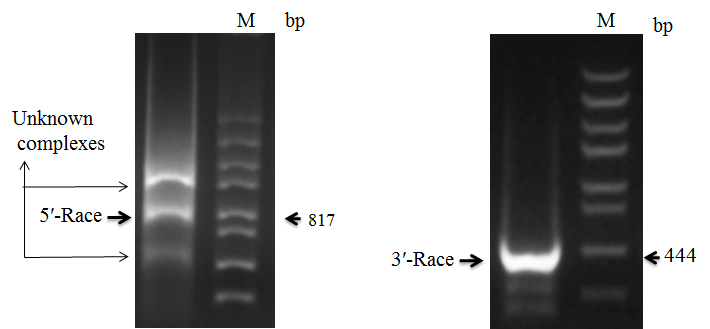


**Figure S1** | The obtained 5ʹ UTR and 3ʹ UTR PCR product of chicken *Dnmt3a* by 5′- and 3′-Race system. Chicken cDNA made from abdominal fat tissue total RNA was used as a template for PCR. Conditions for the PCR were step-down PCR. The PCR products were separated by electrophoresis on a 1.5% agarose gel. M: Marker (Trans5k DNA Ladder, www.transgen.com.cn), 5′ RACE product; 817-bp, 3′ RACE product; 444-bp. The 5′-RACE sequence result is presented in **Figure S2**. The 3′-RACE sequence result was similar to the *Dnmt3a* 3ʹ UTR sequence in GenBank accession No: NM_001024832.1.

**
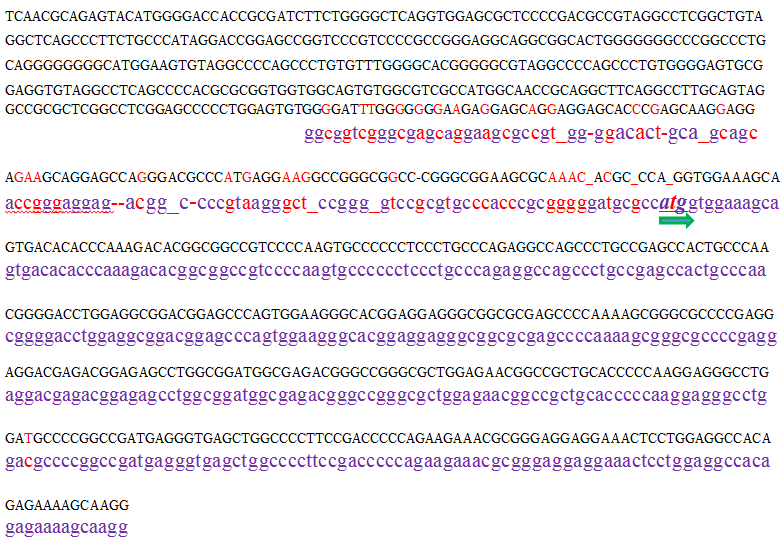
**

**Figure S2** | Sequence comparison between the *Dnmt3a* 5ʹ-Race result (817-bp) and the *Dnmt3a* 5ʹ-UTR (GenBank accession No: NM_001024832.1). Sequence shown in capital letters (black) is a novel transcript’s 5ʹ-UTR of chicken *Dnmt3a* aligned with *Dnmt3a* (GenBank accession No: NM_001024832.1) shown in small letters (purple). The green arrow indicates the start codon of the *Dnmt3a* (NM_001024832.1). Letters highlighted in red represent the differences between two sequences. The black capital letters in top sequence (more than 4 lines) represent additional nucleotides for the novel transcript’s 5ʹ-UTR of chicken *Dnmt3a*. Sequence alignments were performed using the SEQMAN package (DNASTAR Lasergene) software version 7.1.0., and BLAST Nucleotide (GenBank).


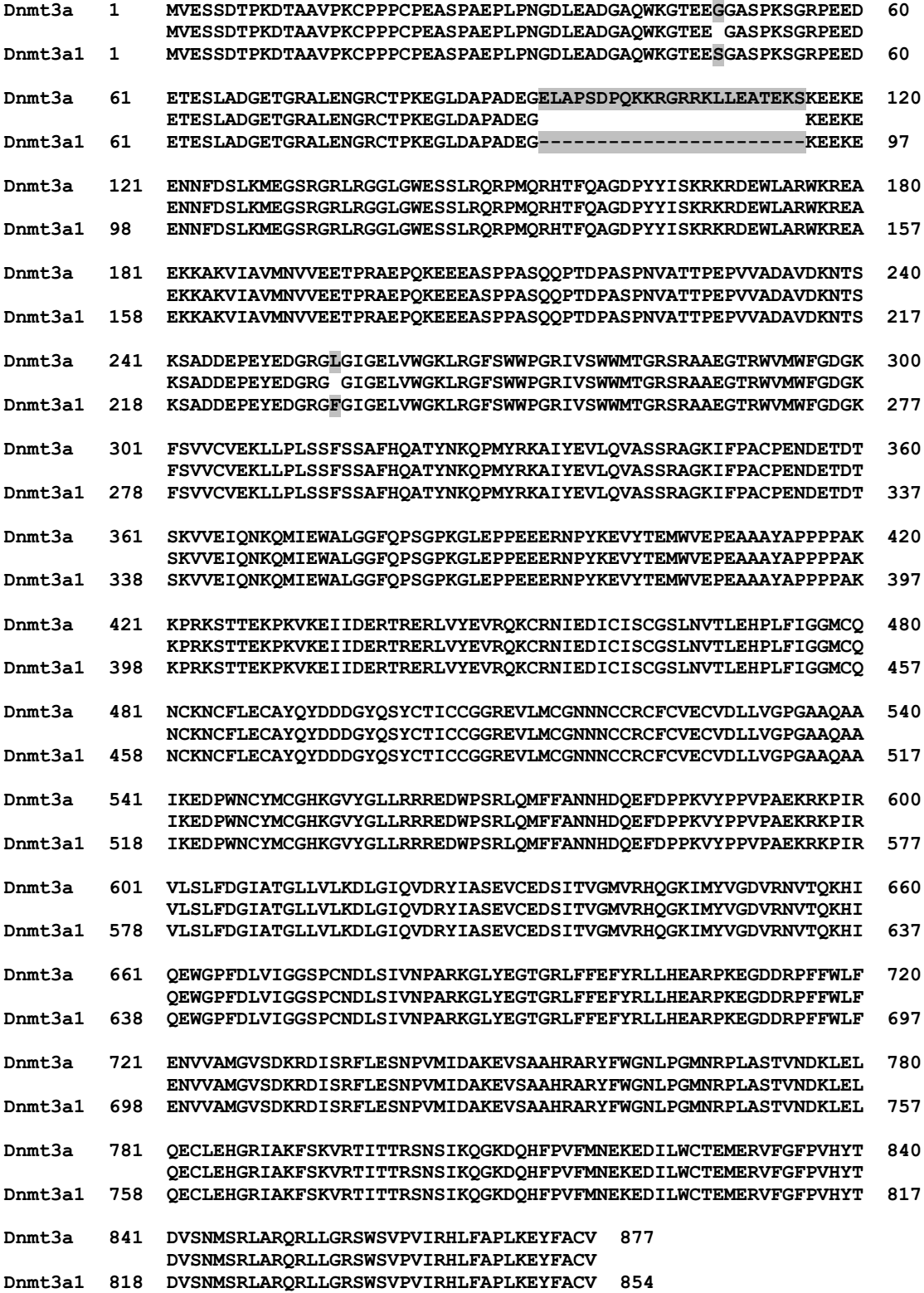


**Figure S3** | The open reading frame (ORF) of chicken Dnmt3a and Dnmt3a1. Dnmt3a1 ORF contains 854 amino acids [DNA Data Bank of Japan (DDBJ) accession No. LC379990], whereas Dnmt3a ORF contains 877 amino acids (DDBJ accession No. LC381635). BLAST was used to analyze the protein homology between Dnmt3a and Dnmt3a1. Letters highlighted in grey indicate either deletion or site of difference in protein sequence.


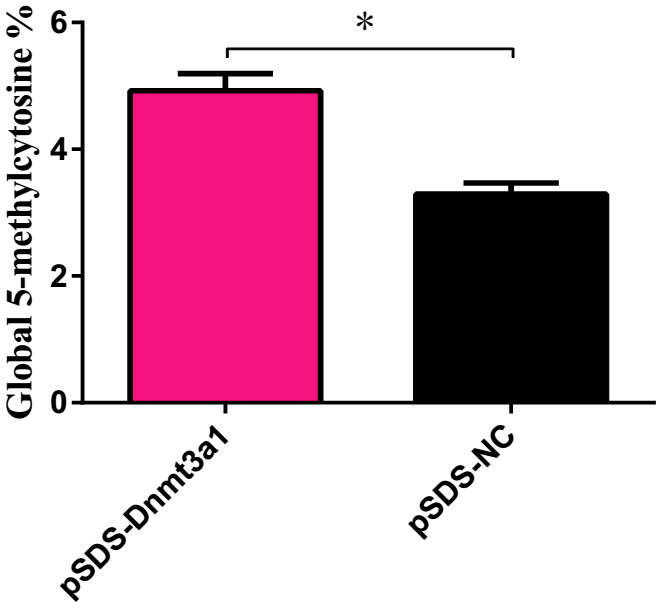


**Figure S4** | Global DNA methylation percentages between *Dnmt3a1* overexpression and the respective negative control in preadipocytes. DNA was extracted from chicken preadipocyte transfected with pSDS-Dnmt3a1 or pSDS-NC, and the global DNA methylation percentages were assessed by using antibody against 5-methylcytosine. % 5-methylcytosine of three independent experiments was quantified. Error bars represent ± SEM for triplicate experiments.


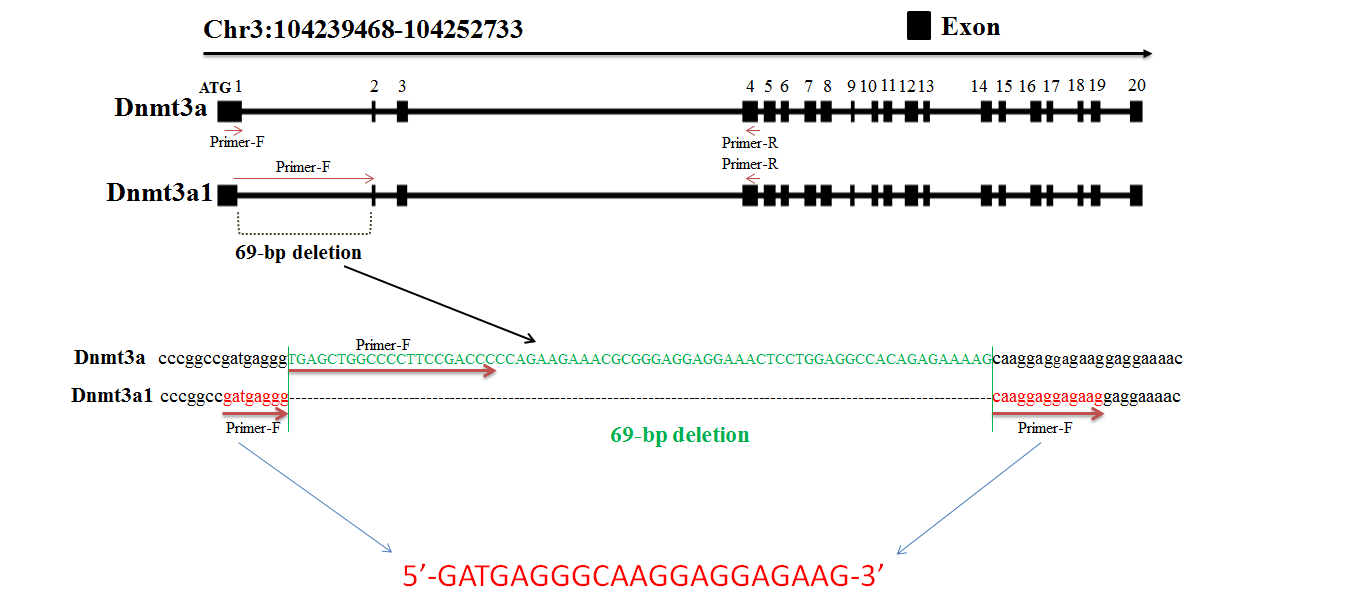


**Figure S5** | qRT-PCR primers at the specific locations used to differentiate the expression of *Dnmt3a* and *Dnmt3a1*. Structure of chicken two variant transcripts (*Dnmt3a* and *Dnmt3a1*) of *Dnmt3a* is shown. Exons are described by closed squares. Letters highlighted in red indicate the location of forward primer (Primer-F) of *Dnmt3a1*. Red arrows indicate the location of primers (*Dnmt3a* and *Dnmt3a1*)*.* The deleted sequences (69-bp) in *Dnmt3a1* were shown in green. The *Dnmt3a1* and *Dnmt3a* qRT-PCR primer sequences (see **Table 3)** were designed by Premier Primer 5.0 Software or Oligo Primer Analysis Software v. 7, and synthesized by Tsingke Biological Technology.


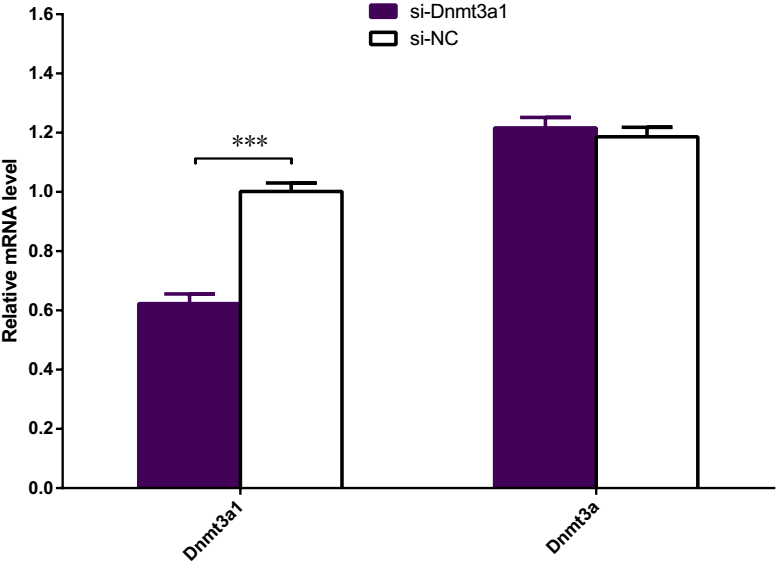


**Figure S6** | si-Dnmt3a1 specifically targeted chicken *Dnmt3a1* mRNA in preadipocytes. Total RNA was extracted (see MATERIALS AND METHODS) from chicken preadipocyte transfected with si-Dnmt3a1 or si-NC, and the mRNA levels of *Dnmta1* and *Dnmt3a* were evaluated by qRT-PCR analysis using primers listed in **Table 3**. mRNA level of three separate experiments was quantified. Error bars represent ± SEM for triplicate experiments. ****P* < 0.001.


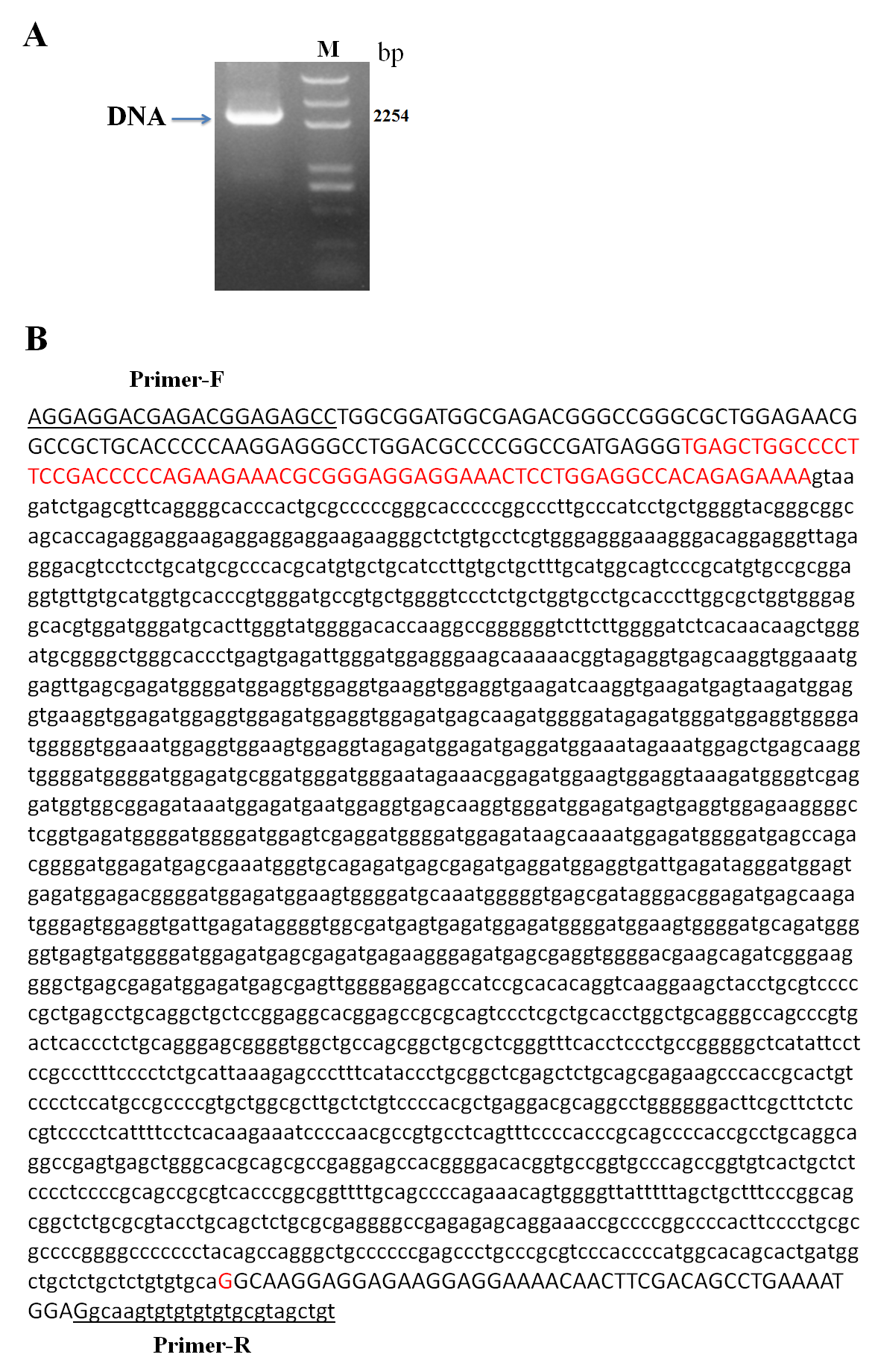


**Figure S7** | Deletion of the 69-bp in *Dnmt3a1* does not exist in genomic DNA level. **(A)** The obtained genomic DNA (PCR product) of chicken *Dnmt3a* by PCR analysis. The genomic DNAs from abdominal fat tissues of 3-wk-old female Guangxi three-yellow chickens (n = 4) were extracted by E.Z.N.A^®^ Tissue DNA Kit-cultured cell protocol (OMEGA Bio-Tek, USA) following the supplier’s recommendations. About 100-ng of genomic DNA was used as a template for PCR analysis performed with the PCR KOD FX reagents (Toyobo Life Science Department, Japan) according to the manufacturer’s instructions. The primer pair [designed from the *Dnmt3a* gene (GenBank accession No: NM_001024832.1)] used to amplify the fragment (2254-bp) of chicken *Dnmt3a* gene containing the deleted area of *Dnmt3a1* is listed in **Table 1**. Conditions for the PCR were: predenaturation for 2 min at 94°C, followed by 38 cycles of denaturation at 98°C for 10 s, annealing at 67 °C for 30 s and extension at 68°C for 2 min, and finally extension at 68°C for 7 min. The PCR products were separated by electrophoresis on a 1.5% agarose gel. M: Marker (*Trans*2k Plus DNA Ladder (5000-bp in size), www.transgen.com.cn), DNA PCR product; 2254-bp. **(B)** The *Dnmt3a* DNA PCR product sequence result. Sequence shown in capital letters (black or red; *top sequence*) is an exon-1 (not whole) of chicken *Dnmt3a.* The chicken *Dnmt3a* intron-1 is shown in small letters in the middle of the sequence. The chicken *Dnmt3a* intron-2 (small part) is shown in small letters *underlined* in bottom sequence. The black capital letters with one nucleotide (red) in bottom sequence represent exon-2 of *Dnmt3a*. The *underlined* sequence indicates the location of forward primer (primer-F) or the reverse primer (primer-R) of *Dnmt3a* used in this analysis. Letters highlighted in red represent the location of the 69-bp deleted sequence at the exon-1/exon-2 border in *Dnmt3a1*. The amplified product was sequenced by Tsingke Biological Technology. Sequence analysis was performed by the SEQMAN package (DNASTAR Lasergene) software version 7.1.0., and BLAST Nucleotide (GenBank). Sequencing confirmed that the 69-bp deletion in *Dnmt3a1* does not exist in genomic DNA level. The experiment was repeated at least 3 times.

Top of Form

|  |
| --- |

Bottom of Form

Top of Form

Bottom of Form

Top of Form

Bottom of Form

Top of Form

Bottom of Form

Top of Form

Bottom of Form

Top of Form

Bottom of Form

Top of Form

Bottom of Form

Top of Form

Bottom of Form

Top of Form

Bottom of Form
